# The genotype-phenotype map of an evolving digital organism

**DOI:** 10.1371/journal.pcbi.1005414

**Published:** 2017-02-27

**Authors:** Miguel A. Fortuna, Luis Zaman, Charles Ofria, Andreas Wagner

**Affiliations:** 1 Department of Evolutionary Biology and Environmental Studies, University of Zurich, Zurich, Switzerland; 2 Department of Biology, University of Washington, Seattle, Washington, United States of America; 3 BEACON Center for the Study of Evolution in Action, Michigan State University, East Lansing, Michigan, Washington, United States of America; 4 Department of Computer Science and Engineering, Michigan State University, East Lansing, Michigan, Washington, United States of America; 5 Swiss Institute of Bioinformatics, Lausanne, Switzerland; 6 The Santa Fe Institute, Santa Fe, New Mexico, Washington, United States of America; University of Texas at Austin, UNITED STATES

## Abstract

To understand how evolving systems bring forth novel and useful phenotypes, it is essential to understand the relationship between genotypic and phenotypic change. Artificial evolving systems can help us understand whether the genotype-phenotype maps of natural evolving systems are highly unusual, and it may help create evolvable artificial systems. Here we characterize the genotype-phenotype map of digital organisms in Avida, a platform for digital evolution. We consider digital organisms from a vast space of 10^141^ genotypes (instruction sequences), which can form 512 different phenotypes. These phenotypes are distinguished by different Boolean logic functions they can compute, as well as by the complexity of these functions. We observe several properties with parallels in natural systems, such as connected genotype networks and asymmetric phenotypic transitions. The likely common cause is robustness to genotypic change. We describe an intriguing tension between phenotypic complexity and evolvability that may have implications for biological evolution. On the one hand, genotypic change is more likely to yield novel phenotypes in more complex organisms. On the other hand, the total number of novel phenotypes reachable through genotypic change is highest for organisms with simple phenotypes. Artificial evolving systems can help us study aspects of biological evolvability that are not accessible in vastly more complex natural systems. They can also help identify properties, such as robustness, that are required for both human-designed artificial systems and synthetic biological systems to be evolvable.

## Author summary

The phenotype of an organism comprises the set of morphological and functional traits encoded by its genome. In natural evolving systems, phenotypes are organized into mutationally connected networks of genotypes, which increase the likelihood for an evolving population to encounter novel adaptive phenotypes (i.e., its evolvability). We do not know whether artificial systems, such as self-replicating and evolving computer programs—digital organisms—are more or less evolvable than natural systems. By studying how genotypes map onto phenotypes in digital organisms, we characterize many commonalities between natural and artificial evolving systems. In addition, we show that phenotypic complexity can both facilitate and constrain evolution, which harbors lessons not only for designing evolvable artificial systems, but also for synthetic biology.

## Introduction

In natural and artificial systems that undergo Darwinian evolution by random mutation and selection, a central distinction is that between a genotype (the entire set of genetic material or a digital organism’s set of instructions, respectively) and a phenotype (the set of observable traits encoded by the genotype). This distinction is important for two main reasons. First, genotypic change causes heritable variation, whereas the phenotypic change it brings forth is the substrate of natural selection. Second, phenotypes form through complex processes such as protein folding and embryonic development. These processes influence how genotypic variation is translated into phenotypic variation. Specifically, they influence what kind of variation becomes available to natural selection. They thus also constrain the directions of evolutionary change. Most importantly, they affect the likelihood that new and beneficial phenotypes—evolutionary adaptations and innovations—originate in the first place [1–5].

To understand the biases and constraints in the production of novel phenotypes, it is necessary to understand how genotypic change translates into phenotypic change. The concept of genotype-phenotype mapping was introduced by Pere Alberch in 1991 as a framework for integrating genetics and developmental biology [6]. However, there is no universal definition of the genotype-phenotype map. We refer to the genotype-phenotype map of natural systems as a (mathematical) function from a space of genotypes to a space of phenotypes, which determines how genotypic information specifies phenotypes through processes such as protein folding and embryonic development. Genotype-phenotype maps have been studied in multiple biological systems, including proteins and RNA molecules [7–12], genome-scale metabolism [13, 14], as well as biological circuits that regulate gene activity [15–17]. These studies have revealed a number of commonalities among otherwise very different systems. One of them is that such systems are to some extent robust to genotypic change [7–21]. Another is that this robustness leads to the existence of genotype networks [9, 10, 14, 15, 17, 22, 23], i.e., networks of genotypes that share the same phenotype, and that can be converted into one another by a series of phenotype-preserving small genetic changes (point mutations). Such networks can facilitate the origins of novel phenotypes because they help populations explore many different regions in genotype space that may harbor such phenotypes [24–26]. A third commonality is pervasive epistasis—non-additive interactions among individual mutations—which makes the phenotypic effects of individual mutations highly dependent on the genetic background on which they occur [27–29].

In addition to naturally evolving systems, researchers are exploring an increasing number of synthetic or artificial evolving systems [30–36] that range from minor modifications of natural systems, such as proteins with non-natural amino acids [37–41], to completely artificial systems such as digital organisms and computer viruses [31, 33, 34]. We know little about the genotype-phenotype maps of such artificial systems. Specifically, we know almost nothing about the organization of their genotype spaces, and how readily novel adaptive phenotypes can originate in such spaces. Such knowledge may help us compare and contrast natural and artificial evolving systems, including the extent to which natural systems are more evolvable. Any such comparison should take into account that the genotype-phenotype map of artifical systems has not evolved, but in contrast to that of natural systems, is designed. Here we address these issues with the Avida platform for digital evolution [30].

**Fig 1:**
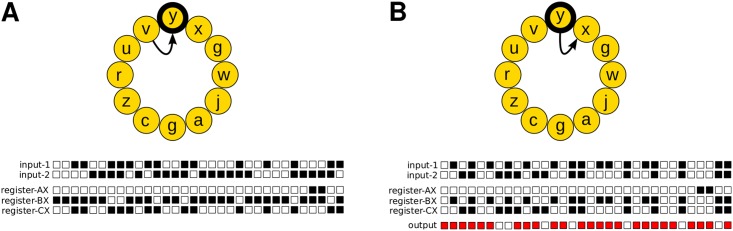
The genotype encodes the phenotype of a digital organism. The genotype of a digital organism with the smallest genome required to perform the logic operation NAND is depicted as a circular set of 12 instructions (represented here as letters). Beyond the instructions necessary for copying the genome, the genetic language of Avida contains instructions for storing and manipulating 32-bit binary numbers in buffers (input-1 and input-2) and registers (AX, BX, and CX). Each binary number is represented here as a sequence of 32 boxes, one for each bit. The value of each bit is depicted as a black box if it equals one and as a white box if it equals zero. The cartoon shows the execution of the input-output instruction (represented by the letter *y*; highlighted in black). (*A*) The state of the input buffers and registers before executing the input-output instruction (the arrow points toward the next instruction to be executed). (*B*) The state of the input-buffers, registers, and the output after executing the input-output instruction. The input-output instruction outputs the number stored in the BX register, checking for any logic operation that may have been performed on the two binary numbers previously stored in the input buffers. In this example, the output is the result of applying the logic operation NAND: for each bit pair, the result is 0 (white box) if and only if the two bits are 1, and 1 otherwise (red box). Then, the input-output instruction places a new random binary number into the BX register (a number that is also stored in the input-1 buffer after moving the number previously stored there to the input-2 buffer). The complete step-by-step self-replication cycle of this digital organism is shown as S1 Appendix. Note that, in our study, the genome of digital organisms is much larger (i.e., 100 instructions long).

Digital evolution is a form of evolutionary computation in which self-replicating computer programs—digital organisms—evolve within a user-defined computational environment [31–33]. Avida is the most widely used software platform for research in digital evolution [33]. It satisfies the three essential requirements for evolution to occur: replication, heritable variation, and differential fitness. The latter arises through competition for the limited resources of memory space and central processing unit (CPU) time. A digital organism in Avida consists of a sequence of instructions—its genome or genotype—and a virtual CPU, which executes these instructions. Some of these instructions are involved in copying an organism’s genome, which is the only way the organism can pass on its genetic material to future generations. To reproduce, a digital organism must copy its genome instruction by instruction into a new region of memory through a process that may lead to errors (i.e., mutations). A mutation occurs when an instruction is copied incorrectly, and is instead replaced in the offspring genome by an instruction chosen at random (with a uniform distribution) from a set of possible instructions. Some instructions are required for replication (i.e., viability), whereas others are required to complete computational operations (such as addition, multiplications, and bit-shifts), and are executed on binary numbers taken from the environment through input-output instructions. When the output of processing these numbers equals the result of a specific Boolean logic operation, the digital organism is said to have a functional trait represented by that logic operation (Fig 1). An organism can be rewarded for having a functional trait with virtual CPU-cycles, which speeds up its execution of instructions. These rewards create an additional selective pressure (besides streamlining replication) which favours those organisms with mutations that have produced sequences of instructions in their genomes that encode functional traits. Organisms that are more successful—those that replicate faster—are more likely to spread through a population.

We use the Avida framework to characterize the genotype-phenotype map of its digital organisms, where this mapping is defined by a direct relationship between complex interactions among computer instructions and the ability for digital organisms to perform Boolean operations. On the one hand, we find that some properties of these maps resemble those found in natural systems, such as robustness, epistasis, and genotype networks. On the other hand, we also characterize a property that has not been found in natural systems. That is, a relationship between phenotypic complexity and the ability to bring forth novel phenotypes [42]. This property may be present but hidden in natural systems, whose overwhelming complexity hinders the analysis of their genotype-phenotype maps. Digital organisms have thus helped us identify a novel hypothesis about the evolvability of natural systems, potentially leading to new fundamental biological principles.

## Results

The genotype space for digital organisms with a genome length (number of instructions) *L* taken from an alphabet of available instructions *A* comprises *A*^*L*^ different genotypes. We here consider genotypes with *L* = 100 instructions drawn from an alphabet of *A* = 26 instructions (Methods), which yields a genotype space of

$$\begin{array}{c} G=26^{100}\approx 3.14\times 10^{141} \end{array}$$ (1)

different genotypes. A genotype in this space encodes a viable organism if it is capable of self-replication. In addition to being viable, the instructions in an organism’s genome may enable it to compute one or more Boolean logic operations. We refer to this ability as a functional trait or as the organism’s phenotype (Fig 1). Specifically, we here focus on 9 logic operations such as the AND and OR Boolean functions, that organisms can perform on 32-bit one- and two-input numbers taken from the environment (Methods). Because any organism could in principle be capable of computing any subset of these operations, the total number of possible phenotypes, i.e., the size of phenotype space, equals 2^9^ = 512 phenoypes. We note that this number includes organisms that are merely viable, i.e., they do not have any functional trait because they cannot perform any of the operations consider in this study.

In a first analysis, we wished to determine the fraction of viable genotypes. To this end, we uniformly sampled genotypes from genotype space until we had found 1000 viable organisms (Methods). This required us to sample 1.5 × 10^9^ genotypes, which implies that the fraction of viable genotypes is ≈1000/(1000 + 1.5 × 10^9^) = ≈ 6.6 × 10^−7^, and its absolute number is ≈ 5 × 10^135^. Because there can be only 512 phenotypes, this result implies that, on average, an astronomical number of genotypes must map onto any of these few possible phenotypes.

**Fig 2:**
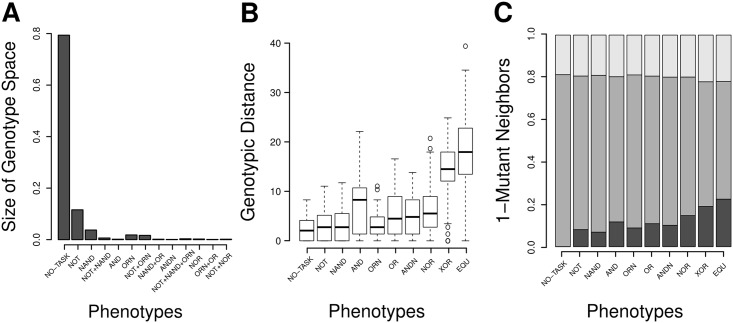
Genotype space characterization. *(A)* A measure of the fraction of viable genotype space (see Methods) in the neighborhood of 1000 merely viable genotypes. We computed the number of 1-mutant neighborhoods of merely viable organisms, in which a particular phenotype (including the merely viable) appeared at least once, divided by 1000, i.e., by the total number of neighborhoods examined. We then normalized this quantity so that the sum equals one. Few phenotypes (e.g., that of merely viable organisms and of organisms able to perform the NOT operation) are moderately frequent, whereas most others (e.g., the NOR phenotype) are rare. *(B)* Genotypic distances between 100 pairs of genotypes per phenotype, after random walks aiming to reach one genotype from the other through multiple phenotype-preserving point mutations. Distance was measured as the number of positions at which both genotypes differ (Hamming distance). *(C)* Classification of genotypes that lie in the 1-mutant neighborhood of every organism having a particular phenotype (x-axis). Each bar shows the fraction of those genotypes that are non-viable (light gray), viable having the same phenotype as the focal phenotype (dark gray), and viable but having a distinct phenotype (black). Phenotypes are arranged from left to right in order of increasing complexity. Panels *B-C* are focused on single-trait phenotypes (i.e., phenotypes whose organisms have only the functional trait posed by a single logic operation) as well as merely viable organisms (labeled as no-trait).

Because not a single genotype in our sample of 1000 viable genotypes was able to compute any logic operation, we wanted to know next whether some of the immediate (1-mutant) neighborhoods of genotypes in this sample have this ability. To this end, we created all *L* × (*A* − 1) = 2500 1-mutant neighbors for each of the 1000 genotypes in our sample, and evaluated the phenotypes of the resulting 2.5 × 10^6^ organisms. Even among this large number of organisms, we found only 13 distinct phenotypes. The proportion of the 1000 neighborhoods in which a phenotype appears at least once indicates a highly non-uniform distribution of phenotypes in genotype space (S1 Fig). These observations suggest that some phenotypes—those we found—are frequent, whereas others must be very rare (Fig 2A). In addition, rarer phenotypes are more complex (*ρ* = −0.759, *n* = 13, *p* = 0.002). We define overall phenotypic complexity as the sum of the complexity of the logic functions that an organism can compute. We approximate each function’s complexity as the minimum number of times that a *nand* instruction—the only instruction that is itself a logic operator—must be executed for computing the function [43, 44]. To compute phenotypic complexity, we add the complexity value of the individual functions, and normalize the resulting sum by the complexity of the most complex phenotype. This measure of phenotypic complexity is not only simple but also sensible: when computed for all 511 functions, it is correlated with the minimum number of times that the *nand* instruction is executed (*ρ* = 0.536, *n* = 511, *p* < 0.001). Note that a complex phenotype results from executing a repeated combination of instructions that simpler phenotypes might already harbor in their genomes.

Given the low number of phenotypes our random sampling had identified, we next undertook a two-step procedure to sample genotypes with all 512 phenotypes (directional selection followed by purifying selection; see Methods). Briefly, the first step consisted of evolving 1000 populations of digital organisms subject to repeated cycles of mutations and selection for specific functional traits (i.e., favoring organisms with genomes where mutations had produced sequences of instructions that compute specific logic operations). We initialized each population from one of the 1000 randomly sampled viable genotypes. We allowed these 1000 populations to evolve for 10^6^ updates, where an update is the amount of time during which an organism executes on average 30 instructions. After 10^6^ updates, the total number of distinct phenotypes encountered in each evolving population did not increase further. At that point in time, we stopped the evolution process and kept only one genotype per phenotype, chosen at random from the genotypes previously encountered during the process. This procedure allowed us to find at least one genotype that mapped to each one of the 512 phenotypes comprising the whole phenotype space. We observed that 60% of phenotypes were discovered by only 10% of the populations, and only 12% of phenotypes were found by more than 90% of the populations (S2 Fig). A few phenotypes—likely the rarest ones—were very difficult to find, and two of them were discovered by only one population.

In the second step, we aimed to obtain a fixed number of 1000 independently sampled genotypes for each phenotype. To this end, we started from the previously discovered genotypes with a specific phenotype, and performed double-mutant random walks through genotype space that preserved viability and phenotype during 1000 mutational steps (Methods). For each phenotype, we performed 1000 such random walks, thus creating 1000 randomly sampled genotypes with this phenotype (data are provided as S1 File).

With these samples in hand, we first asked how different two organisms can be in their genotypes if they share the same phenotype. For every phenotype, we found that the maximum genotype distance—measured as the Hamming distance of genotype instruction sets—among all genotypes with the same phenotype is as high as the genome length, *D* = 100. That means that organisms whose genotypes differ in all positions along their genomes may indeed have the same phenotype (Methods). This is possible because the effect of an instruction on a phenotype depends on other instructions contained in the genome, a phenomenon analogous to epistasis in genetic systems [43]. By applying a multiple local alignment algorithm to the genomes of organisms with the same phenotype (Methods), we did not find any recurring subsequence pattern—sequence motif—revealing common ways of achieving the same phenotype nor any sequence motif containing the instructions required for viability. We only found a small motif in genotypes encoding the simplest phenotypes. It contains the flow-control operations involved in determining which intructions are going to be read and written (S3 Fig). Overall, these observations show that genotypes with any one phenotype are not localized in a single small region of genotype space, but might rather occur throughout this space.

We next asked whether genotypes with the same phenotype can be connected in sequence space through a series of point mutations (single instruction changes) that leave the phenotype intact. In other words, do genotypes with the same phenotype form a single connected network of genotypes? To find out, we performed random walks involving multiple pairs of genotypes with the same phenotype, where each random walk aimed to reach one of the genotypes from the other without changing its phenotype. We found that this is not generally possible, and recorded the minimal distance between genotypes that we were able to obtain with this approach (Methods). Since this is a computationally time-consuming process, we carried it out only for merely viable organisms and for organisms with single-trait phenotypes. We note that the random walks we performed can only provide upper bounds on the distance between different components of the same genotype network. Fig 2B shows the minimal distances between pairs of genotypes for single-trait phenotypes, arranged as a function of the complexity of the trait. We found that at least one pair of genotypes of every phenotype is connected and that the average minimum distance between genotype pairs increases with trait complexity (*ρ* = 0.940, *n* = 10, *p* = 0.005, for the median).

Even though the preceding observations suggest that genotype network fragmentation rises with phenotypic complexity, an additional analysis shows that the gaps between different genotype networks might be easily bridged. In this analysis, we performed random walks analogous to those just described, where each step needed to preserve both viability and the phenotypic traits of the starting genotype. In addition, we also accepted steps that lead to genotypes with additional traits that had not been present in the starting genotype. Under these conditions, the average minimum distance between pairs of genotypes became significantly lower than in the preceding analysis (from 4.5 to 4 for the simplest phenotype, and from 25.5 to 18 for the most complex one). In addition, the fraction of genotype pairs that were connected increased by 11% for the simplest single-trait phenotype, and by 63% for the most complex one. Because the additional traits we observe were not required by our selection criterion, they emerged spontaneously. In the language of evolutionary biology, they can thus be viewed as potential exaptations [44]—traits of organisms that are either not adaptive when they originate, or whose adaptive role changes [45].

Different phenotypes may not only differ in the number of genotypes that form them. They may also differ in their accessibility from genotypes with other phenotypes, that is, in the likelihood to reach them from such a genotype through a single point mutation. To estimate such differences in phenotypic accessibility, we used our samples of 1000 organisms with a given phenotype, and computed, for all phenotypes *i* and *j*, the probability of encountering an organism with phenotype *j* from an organism with phenotype *i* by a single point mutation. To this end, we first identified all genotypes that lie in the 1-mutant neighborhood of every organism having phenotype *i*. We then classified these genotypes according to phenotype, and computed the fraction of those genotypes that have phenotype *j*. We also refer to this fraction as the transition probability from phenotype *i* to phenotype *j* (*p*_*i*→*j*_).

The organisms we encountered in these neighborhoods fall into three classes. The first class holds inviable organisms. Fig 2C shows that the likelihood of encountering an inviable organism through a point mutation (*p*_*i*→0_) increases with the complexity of the phenotype *i* (Spearman’s *ρ* = 0.621, *n* = 512, *p* < 0.001; S4 Fig).

The second class comprises viable organisms that have the same phenotype as *i* (*p*_*i*→*i*_, see Fig 2C). We refer to the fraction of point mutations that preserve an organism’s phenotype, averaged over all organisms with this phenotype, as the mutational robustness of this phenotype. The higher the complexity of a phenotype, the lower is its robustness (*ρ* = −0.689, *n* = 512, *p* < 0.001; S4 Fig). Since some instructions of an organism’s genome might not be executed during its self-replication process, we asked to what extent simple phenotypes correspond to organisms that execute fewer instructions. To answer this question, we computed the fraction of the genome as well as the number of instructions that organisms encoding the same phenotype executed during their replication. Neither one nor the other were correlated with phenotypic complexity (*ρ* = 0.017, *n* = 512, *p* = 0.709; *ρ* = −0.049, *n* = 512, *p* = 0.271; respectively). Another factor that could be responsible for the association between phenotypic complexity and robustness is the fixed length of the genome. To rule this possibility out, we reduced the genome size for all organisms with single-trait phenotypes by deleting one randomly chosen instruction at a time, while preserving viability and phenotype, until no more instructions could be removed. We found that organisms having more complex functional traits required a larger minimal genome (*ρ* = 0.902, *n* = 9, *p* < 0.001, for the median, see S5 Fig). This observation implies that the higher the phenotypic complexity of an organism is, the smaller is the number of instructions in the genome that can be altered without perturbing the phenotype. In other words, phenotypic complexity comes at the price of lower phenotypic robustness.

**Fig 3:**
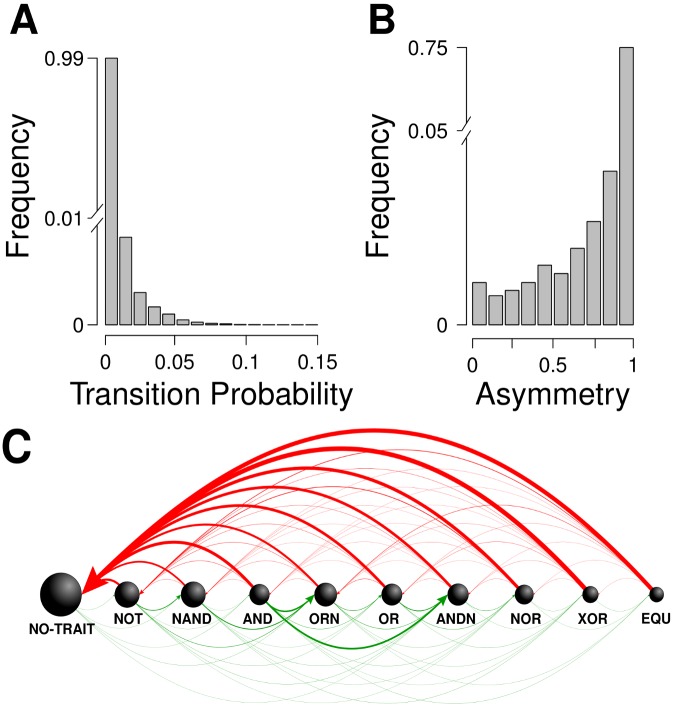
Most phenotypic transitions are rare and asymmetric. *(A)* Distribution of transition probabilities from phenotype *i* to any other phenotype *j*, computed as the fraction of all genotypes with phenotype *j* that lie in the 1-mutant neighborhoods of organisms with phenotype *i*. Most transition probabilities are very low. *(B)* Distribution of the asymmetry *AS*(*i*, *j*) of the transition probabilities between all pairs of phenotypes *i* and *j*. Most reciprocal transition probabilities are highly asymmetric. *(C)* Transitions between single-trait phenotypes (i.e., phenotypes whose organisms have only one single trait) as well as merely viable organisms (labeled as no-trait). Nodes represent phenotypes (arranged in order of increasing complexity from left to right) and arrows depict transition probabilities. Node size is scaled to the logarithm of phenotypic robustness (i.e., the fraction of 1-mutant neighbors without altered phenotype). Transitions from phenotype *i* to phenotype *j*, where *j* is more (less) complex than *i* are depicted by green (red) arrows. The thickness of an arrow between two nodes is proportional to the transition probability between the phenotypes represented by that pair of nodes. (Green arrows are drawn 10 times thicker than red ones for visualization purposes.). The figure illustrates that (i) it is generally harder for a simple phenotype *i* to reach a more complex phenotype *j* than vice versa; (ii) the only way to encounter the most complex single-trait phenotype (EQU) from the least complex one of mere viability (bottom) requires going through at least two phenotypes of intermediate complexity (e.g., to NOT, AND, and from there to EQU).

The third and most important class of organisms comprises the 1-mutant neighbors that have a different phenotype *j*. The greater the complexity of phenotype *i* is, the greater is the probability (*p*_*i*→*j*_) of finding a genotype with a novel phenotype (*ρ* = 0.633, *n* = 512, *p* < 0.001; see Fig 2C and S4 Fig). We also found that the distribution of non-zero transition probabilities (68%) is heavy-tailed (Fig 3A). This means that only a few novel phenotypes are highly accessible through single point mutations, whereas most have a very low chance of being encountered.

The probabilities of phenotypic change may be asymmetric [46, 47]; that is, phenotype *i* may be easily accessible from phenotype *j* but not vice versa (*p*_*i*→*j*_ ≠ *p*_*j*→*i*_). We quantified this asymmetry by computing the quantity *AS*(*i*, *j*) = |*p*_*i*→*j*_ − *p*_*j*→*i*_|/max(*p*_*i*→*j*_, *p*_*j*→*i*_), where max refers to the maximum of two values [48]. We found that most reciprocal transition probabilities are highly asymmetric (Fig 3B). These asymmetries in transition probabilities are just a consequence of the fact that different phenotypes have different numbers of genotypes that code for them (see Methods for a simple mathematical explanation). This direct relationship between transition probabilities and the frequency of phenotypes has also been reported in models for protein folding and self-assembling protein quaternary structure [49]. Indeed, the ratios of the transition probabilities between pairs of phenotypes provide an estimate of the ratios of the frequencies of each phenotype in genotype space (although this estimate might deviate from the exact value because of sampling errors).

We also estimated the frequency of the single-trait phenotypes in genotype space relative to the number of merely viable organisms *N*_*j*_. That is, $N_{i}=\frac{p_{j\to i}}{p_{i\to j}}\times N_{j}$, where *N*_*j*_ = 1. It ranges between 10^−3^ and 10^−11^ for the simplest and most complex phenotypes, respectively. We found a negative relationship between the estimated frequency of each phenotype and its phenotypic complexity (*ρ* = −0.889, *p* = 0.001, *n* = 9). This result explains the association found between phenotypic complexity and phenotypic transition probabilities. Specifically, for 90% of phenotype pairs *i* and *j*, the probability of encountering phenotype *i* from phenotype *j* was higher if *j* was more complex than *i*. In other words, it is harder for a simple phenotype *i* to reach a more complex phenotype *j* than vice versa because genotypes with complex phenotypes are less common than genotypes with simple ones (see Fig 3C). According to the predictions of models assuming a random distribution of genotypes in genotype space [50], the robustness of single-trait phenotypes increases logarithmically with the frequency of the phenotypes estimated from the ratios of their transition probabilities (*R*^2^ = 0.876, *n* = 9, *p* < 0.001).

Computational approaches have shown that epistasis is more common between mutations that fix under purifying selection than among randomly selected mutations [29, 51, 52]. Therefore, our non-uniform sampling procedure to find genotypes encoding the same phenotype (directional selection followed by purifying selection) might influence the topology of the genotype-phenotype map around evolved genotypes. To rule out this possibility, we calculated the correlation between the proportion of the 1000 neighborhoods of the merely viable organisms (randomly sampled) in which a phenotype appears at least once, and the frequencies of those phenotypes estimated from the ratio of the transition probabilities for our evolved genotypes. We found a positive and statistically significant relationship between the two estimates of the size of the genotype space occupied by a given phenotype (*ρ* = 0.985, *n* = 13, *p* < 0.001). This suggests that the topology of the genotype space around evolved genotypes might not be different from that around randomly sampled ones (at least for the single-trait phenotypes).

We next studied the evolvability of individual genotypes with phenotype *i*, which we define as the number of distinct phenotypes *j* ≠ *i* that can be reached by a single point mutation from genotypes with phenotype *i*. This *genotypic* evolvability increases with phenotypic complexity (*ρ* = 0.833, *n* = 511, *p* < 0.001). This association might be a simple consequence of the fact that it is easier to lose abilities (functional traits) than to gain them by random mutation. To exclude such degenerative mutations, we repeated this analysis with a constrained definition of evolvability including only those phenotypes *j* as novel that can compute at least one additional logic function compared to *i*. Because the number of phenotypes with novel traits *j* ≠ *i* decreases as the complexity of phenotype *i* increases, we divided the evolvability of phenotype *i* by the total number of phenotypes with novel traits *j* ≠ *i*. Even with this much more conservative notion of evolvability, genotypes with more complex phenotypes were more evolvable (*ρ* = 0.832, *n* = 510, *p* < 0.001).

**Fig 4:**
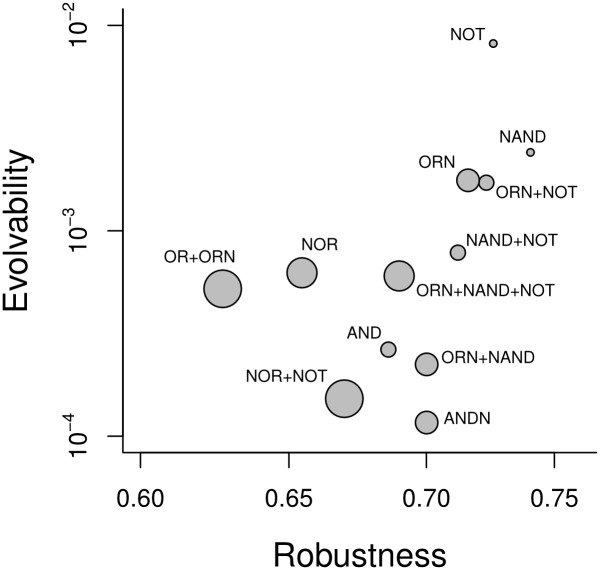
Phenotypic evolvability increases with phenotypic robustness. We computed the robustness of phenotype *i* as the fraction of 1-mutant neighbors with the same phenotype as *i*, averaged over the 1000 genotypes with phenotype *i*. We computed the evolvability of phenotype *i* as the fraction of the phenotype space that occur in the 1-mutant neighborhood of 1000 genotypes with phenotype *i*, multiplied by the frequency of phenotype *i*. Only those phenotypes where we have frequency data are considered in this figure. Text labels indicate the logic functions that define each phenotype. The diameter of the circles is proportional to phenotypic complexity.

The preceding analysis did not take into account that different phenotypes differ in the size of their genotype network. That is, we analyzed the same number of genotypes for each phenotype, regardless of the fraction of genotype space occupied by each phenotype. This approach can be biased because genotype network size can affect the total number of novel phenotypes that are reachable by one mutation from *any* genotype with a given phenotype [53]. We refer to this number also as the evolvability of a *phenotype*, as opposed to that of a genotype. In other words, rare phenotypes were sampled more intensively than common ones. To estimate this phenotypic evolvability, we multiplied the genotypic evolvability from the preceding paragraph by the frequency of the corresponding phenotype in genotype space, which adjusts for genotype network size assuming that the number of phenotypes found scales linearly with the number of genotypes sampled. Fig 4 shows that evolvability increases with robustness for the 13 phenotypes for which we have frequency data (*ρ* = 0.754, *n* = 13, *p* = 0.003). In addition, more complex phenotypes (larger circles) are less evolvable (*ρ* = −0.701, *n* = 13, *p* = 0.008), most likely because they occupy a smaller subset of genotype space.

## Discussion

**Fig 5:**
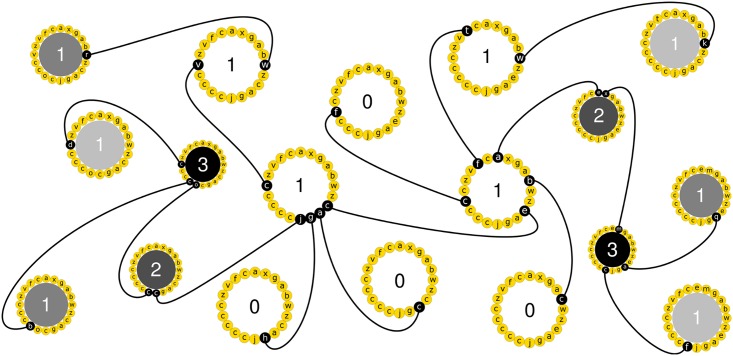
Cartoon summarizing the architecture of the genotype-phenotype map. This subset of a hypothetical genotype space shows 18 genotypes (large circles). The genotype of each organism is represented by a circular set of 20 instructions (small letters inside small yellow circles). Two genotypes are connected by a black line if they differ in a single instruction (white letters inside small black circles). Only the 1-mutant neighbors that are relevant for characterizing the genotype-phenotype map are drawn. The size of the circle representing an organism’ genotype is proportional to the organism’s robustness to mutations (i.e., to single instruction changes). Phenotypic complexity of each genotype is indicated by gray shading that ranges from white (least complex) to black (most complex). Genotypes with the same phenotype are represented by the same shading. The number of novel phenotypes encoded by the 1-mutant neighbors of each genotype is indicated inside the large circles. The cartoon illustrates several of our main observations. First, the most robust phenotype (largest circles) is the most abundant, and its genotypes likely form a single genotype network (i.e., all pairs of such genotypes can be connected in genotype space through a series of point mutations that leave the phenotype intact). Second, the more complex the phenotype of an organism is (the darker the shading) the larger is its genotypic evolvability (i.e., the number of its 1-mutant neighbors with novel phenotypes), and the smaller its robustness (i.e., the number of its 1-mutant neighbors with the same phenotype). Third, organisms with the least complex phenotype (white circles) can only access the most complex phenotypes (e.g., black circles) through phenotypes of intermediate complexity (gray circles).

Our analysis of the genotype-phenotype map in the artificial life system Avida (see Fig 5) revealed that the number of genotypes forming a phenotype differs greatly among phenotypes. The more complex the logic operations are that a phenotype performs, the fewer genotypes form this phenotype. Genotypes with any one phenotype tend to form one or more networks whose members are likely connected to one another by series of small genotypic changes that leave the phenotype unchanged, and thus help explore different regions of genotype space. The larger any one such network is, the greater is the number of novel phenotypes that can be reached through single point mutations from its members. We also find that the accessibility of novel phenotypes is highly asymmetric: it is much harder to evolve more complex phenotypes than simpler ones through single point mutations.

One of the obvious parallels between biological systems and Avida is that our digital organisms are to some extent robust to genotypic changes, i.e., to “point mutations” in their instruction sequence. It is this robustness that might give rise to large phenotype-preserving genotype networks [54, 55]. In natural systems, most robustness to mutations is a consequence of the fact that organisms must persist in multiple different environments [55]. In an artificial system like Avida, robustness can be achieved in simple ways, by providing a genome with more instructions than needed, as we did. The resulting excessive genomic size allows more flexibility in tinkering with instructions while preserving a phenotype, which facilitates the origin of novel phenotypes near these genotypes. Observations like this provide guiding principles to design evolvable artificial systems.

The genotype networks we examined are not all connected, and may consist of multiple different components. However, this fragmentation is most pronounced when we require the strict preservation of phenotypes in the random walks that aim to connect different organisms with the same phenotype. During some steps of these random walks, genotypes fortuitously acquire novel computational abilities that they do not require, and if we do not allow such “innovative” steps, some genotype networks are disconnected. If, however, we admit such steps, the chances for all phenotypes we examined to be connected in a single genotype network increases. We view such non-adaptive novel traits, which also exist in metabolic systems [56], as analogous to potential exaptations [44]. They are not adaptive, but could become adaptive in the right environment. Moreover, they can help bridge gaps between disconnected genotype networks, and thus make more genotypes accessible by populations subject to phenotype-preserving point mutation. This additional connectivity, in turn, makes more novel phenotypes accessible that reside near these genotypes.

The asymmetric phenotypic transitions we observe, where the likelihood of reaching phenotype *i* from phenotype *j* through a single point mutation is not equal to the converse probability, also have parallels in natural systems. For example, such asymmetries have been observed in phenotypic transitions between different RNA secondary structures [46, 47, 57]. They also occur in anisotropic morphospaces of paleobiology [58], where a clade’s propensity to vary depends on the direction of phenotypic change. In our study system, asymmetric phenotypic transitions result from the vastly different number of genotypes that encode each phenotype. Some phenotypes, regardless of their adaptive value, are more likely to be “discovered” by evolving populations than others. Because such differences in phenotypic rarity are pervasive in other systems [8, 42, 53, 54, 59, 60], asymmetric transitions are likely to be a universal characteristic of phenotypic evolution. Regardless of their causes, they have practical consequences. For example, they can lead to spurious incidences of convergent evolution, and they can mislead reconstructions of ancestral phenotypes [61].

To our knowledge, the relationship we found between phenotypic complexity and evolvability has not been reported for any natural system. These relationships exist on two levels of organization. The first is that of individual genotypes with a specific phenotype. Mutations are more likely to create novel phenotypes in digital organisms with complex phenotypes. It is not difficult to see why, and we fully expect similar causes to be at work in natural systems. Phenotypes emerge from the coordinated execution of “genetic building blocks”, which are analogous to developmental processes guided by regulatory programs in biology. These building blocks can be modified to perform different logic operations. Evolving genomes can “discover” complex phenotypes only by combining the genetic building blocks of preexisting, simpler phenotypes [62]. The genome of organisms with complex phenotypes is expected to harbor more such building blocks, which can be altered and combined in more ways than in organisms with simpler phenotypes.

The second level of organization is that of the entire genotype space. Here, we observe that complex phenotypes are more rare, that is, they are encoded by fewer genotypes. (The larger minimal genomes required for such phenotypes are consistent with this observation, because they constrain genotypic evolution to a smaller region of genotype space). The main consequence of the rarity of complex phenotypes is that the total number of novel phenotypes from any of these genotypes—phenotypic evolvability—is lower.

Both levels of organization can help explain the relationship of asymmetric transitions to phenotypic complexity, i.e., single mutations from any one phenotype are more likely to yield a simple phenotype than a complex one, and the most complex phenotypes can be reached only through multiple steps. From the individual, mechanistic perspective, mutations are more likely to lead to a loss than a gain of a function in a genetic building block required for a phenotype. They are thus more likely to create a simpler phenotype (or an inviable organism), than a complex phenotype. This asymmetry is also at the core of why the most complex phenotypes must be built in multiple small steps. From the collective, genotype space perspective, mutations are simply less likely to “hit” the smaller target of a complex phenotype with a small genotype network.

We expect that natural systems, which display dramatic differences in the number of genotypes that form specific phenotypes [8, 42, 53, 54, 59, 60] would show a similar relationship between complexity and evolvability. If so, two predictions follow. First, mutations in an evolving population whose members have a complex phenotype are more likely to create novel phenotypes. Second, on long evolutionary time scales, these phenotypes may be less diverse than for organisms with a simpler phenotype.

Our results also have implications for the development of genetic languages in artificial life [63]. Not only can Avida organisms display robustness to mutations, Avida’s genetic language is itself robust to several modifications of the instruction set [64]. Only few modifications, such as the separation of the input and output instruction can alter an organism’s ability to perform logic operations. Future studies may systematically compare different genotype-phenotype maps, and identify those that are most evolvable. Their insights may also guide synthetic biologists in designing genetically engineered devices primed for evolutionary innovation [65].

## Methods

### The genotype of a digital organism

The genome of a digital organism is a circular sequence of instructions taken from a 26-instruction alphabet [33]. It comprises instructions for copying, as well as for completing computational operations (such as additions, subtractions, and bit-shifts), which are executed on binary numbers taken from the environment. The default environment provides the organism with new, random input strings every time an input-output instruction is executed. The genome of a digital organism can harbor one or several input-output instructions that can be executed either only once or many times during the time it takes to generate an offspring. This means that the organism can take input numbers from the environment more than once before replicating and can compute the result of more than one logic operation (see below). Only one instruction from the instruction set is itself a logic operator. This is the *nand* (not-and) instruction, which must be executed in coordination with input-output instructions to perform the NAND logic operation. The *nand* instruction reads in the contents of the BX and CX registers and performs a bitwise NAND operation on them (i.e., it returns 0 if and only if both inputs at the corresponding bit positions are 1, otherwise it returns 1). The result of this operation is placed in the BX register. The *IO* (input-output) instruction takes the contents of the BX register and outputs it, checking it for any logic operations that may have been performed. It will then place a new input into BX (see S1 Appendix). All other logic operations must be performed using one or more *nand* instructions in combination with input-output instructions [33]. Since previous work has shown that organisms with a genome of 83 instructions are able to perform the most complex logic operation we consider here [66], we decided to focus on genomes with L = 100 instructions—a genome size large enough to permit exploration of all phenotypes, but at the same time small enough to be computationally tractable.

### The phenotype of a digital organism

Phenotypes are defined by the combination of the following 9 Boolean logic operations that organisms can perform on 32-bit one- and two-input numbers: NOT, which returns 1 at a bit position if the input is 0 at that bit position, and 0 if the input is 1; NAND, which returns 0 if and only if both inputs at the corresponding bit positions are 1 (otherwise it returns 1); AND, which returns 1 if and only if both inputs are 1 (otherwise it returns 0); OR_N (or-not), which returns 1 if for each input bit pair one input bit is 1 or the other is 0 (otherwise it returns 0); OR, which returns 1 if either the first input, the second input, or both are 1 (otherwise it returns 0); AND_N (and-not), which only returns 1 if for each bit pair one input is 1 and the other input is 0 (otherwise it returns 0); NOR (not-or), which returns 1 only if both inputs are 0 (otherwise it returns 0); XOR (exclusive or), which returns 1 if one but not both of the inputs are 1 (otherwise it returns 0); EQU (equals), which returns 1 if both bits are identical, and 0 if they are different [33]. This logic operations are listed above in order, from least complex to most complex. Here, we define complexity as the minimum number of times that a *nand* instruction—the one required to compute all other logic operations—must be executed for completing a specific logic operation. Specifically, their complexities are 1 (NOT), 1 (NAND), 2 (AND), 2 (ORN), 3 (OR), 3 (ANDN), 4(NOR), 4 (XOR), and 5 (EQU) [33]. We used a test environment provided by Avida to compute the phenotype of each digital organism’s genotype. In such a test environment each organism executes its instructions in isolation until it produces a viable offspring or until a timeout is reached, whichever comes first. We note that it is impossible to determine with certainty whether an organism is able to produce a viable offspring (i.e., its viability), because the number of instructions executed before replicating might be extremely large, for example because they might involve loops. We therefore limit how long an organism remains in the test environment before assuming that it is not going to replicate. Specifically, we set this limit to 20 × L because we found no additional viable organism when a sample of 10^7^ randomly generated genomes was left in the test environment twice as long as our limit. That is, we kept each organism in the test environment until it had executed 2000 instructions. For the purpose of determining an organism’s phenotype, we allowed no mutations, such that the offspring is an exact copy of its parent. We recorded the logic operations performed by the organism in the test environment, thus assigning a unique phenotype to each genotype. Note that we have also explored to what extent a variable environment may elicit additional phenotypes for the same genotype (S6 Fig).

### Sequence motifs

Instruction sequences representing the genomes of digital organisms might contain similar regions (instruction sequence motifs) that reflect similar ways of achieving specific phenotypes and/or self-reproduction. To find out whether such regions exist, we have applied the GLAM2 algorithm [67, 68] for discovering both gapless and gapped motifs from the instruction sequences constituting the genomes of our sampled digital organisms. We searched for overrepresented gapped motifs because digital organisms may execute jump instructions that move the execution flow from one region of the genome to another. Although searching for gapped motifs might miss jumps, it would be less appropriate to search for gapless motifs in Avida. One of the advantages of GLAM2 is that it operates on sequences over arbitrary, user-defined alphabets. GLAM2 defines a scoring scheme for local alignments of multiple sequences and finds the alignment with the maximum score using simulating annealing. Since GLAM2 is a heuristic algorithm, we ran it 100 times to verify that it finds a reproducible, highly-scoring motif (we used the default settings, except very large values for the following parameters to turn off deletions and insertions completely: -E 1e99 -J 1e99). GLAM2 provides the statistical significance of an alignment by comparing its score with that obtained after a random reshuffling of the instructions along the sequences.

### Sampling genotype space

To sample genotype space, we first aimed to generate 1000 viable organisms. To this end we first generated random genomes with 100 instructions, where we chose each instruction in a genome randomly and uniformly among the 26 possible instructions, and examined each genome for viability. After having generated 1.5 × 10^9^ genomes in this way, we had found 1000 viable genomes. None of them were able to perform any logic operation. Next, we evolved 1000 populations of organisms in the standard mode of Avida, where we initialized each of the populations with one of the 1000 previously sampled organisms. We configured the standard mode of Avida to follow a Moran process, where every time an organism produces a viable offspring, the offspring replaces one organism randomly chosen from a population of 10^4^ organisms. In our simulations, each offspring differed from its parent by a single point mutation, i.e., one randomly chosen instruction in its genome was replaced with a instruction randomly chosen from the instruction set. In addition, we rewarded the ability of an organism to perform any of the 9 logic operations defining a phenotype with an extra amount of virtual CPU-cycles that sped up its replication process. This procedure introduced a selective pressure that favored organisms with genomes where mutations had produced sequences of instructions that compute one or more logic operations (the more the better). We let each population evolve for 10^6^ updates, where an update is the amount of time during which an organism executes on average 30 instructions. Every 1000 updates we recorded, for every distinct phenotype encountered in the population at that time, the genotype of one randomly chosen organism with that phenotype. After 10^6^ updates, the number of distinct phenotypes encountered in each evolving population reached an asymptote. Then, we stopped the evolution process and kept only one genotype per phenotype and population, chosen at random from those previously recorded during the process. These 1000 evolving populations were enough to find at least one genotype that mapped to each one of the 512 phenotypes comprising the whole phenotype space. This number of 512 phenotypes includes the phenotype of the ancestors (i.e., merely viable organisms). In order to obtain a fixed number of 1000 independently sampled genotypes for each phenotype, we then performed 1000 random walks through the genotype space for each phenotype. These random walks started from the organisms (genotypes) with a given phenotype that had been found by our evolving populations. Some genotypes were used more than once because for some phenotypes fewer than 1000 genotypes with that phenotype had been found. We performed these random walks in the test environment. Each step in each random walk mutated two randomly chosen instructions in the random-walking genotype, and replaced them with two randomly chosen instructions from the 26-instruction alphabet. Whenever such mutations produced a non-viable organism or an organism whose phenotype had changed, we reverted the mutations and mutated two new, randomly chosen instructions, repeating this procedure until a viable organism with an unchanged phenotype appeared. We repeated this procedure for 1000 steps, that is, until a chain of 1000 viable organisms with the same phenotype as the starting genotype had been discovered, and kept the last genotype in the chain for further analysis. In sum, this procedure helped us create 1000 randomly sampled viable organisms for each phenotype (data are provided as S1 File).

### Searching for genotype networks

We wished to estimate to what extent organisms with the same phenotype are connected in a single network of genotypes (a graph whose nodes are genotypes with the same phenotype and where two nodes are connected if they differ by a single instruction). To this end, we started from 100 pairs of organisms with identical phenotypes produced through the random walks described above. For each such pair, we performed a random walk through genotype space, in which we changed one member of the pair through a series of single point mutations, where each mutation was required to preserve both viability and phenotype. In addition, no mutation was allowed to increase the genotype distance to the second member, which was measured as the number of positions at which the genomes of both organisms differed, i.e., their Hamming distance. The goal of each random walk was to find a path through genotype space that would approach and eventually reach the other member of the pair of genotypes, while preserving the phenotype. Note that our algorithm does not take into account that finding connections among genotypes encoding the same phenotype might require reversals of mutations. After 10^4^ steps, that is, until a chain of 10^4^ viable organisms with the same phenotype as the initial genotype had been discovered, we counted the number of instruction matches in the genome of the random walker and the other member of the initial genotype pair. We repeated this procedure 10 times for each of the 100 pairs of organisms with a given phenotype. Finally, we recorded the smallest distance value from these 10 × 100 = 1000 replicates as the minimum genotype distance between the organisms with the same starting phenotype. This process is computationally time-consuming and we performed it only for the single-trait phenotypes (i.e., those corresponding to a single logic function). In addition, we repeated the entire process by relaxing the criterion of exact phenotype preservation during a random walk. Specifically, in this kind of random walk, the random walkers had to preserve viability and all the logic operations they were able to perform at the beginning of the random walk, but if they acquired the ability to perform additional logic operations during any one step (but not any fewer), we considered that step acceptable.

### Phenotypic transitions

To estimate how likely it is that single point mutations cause transitions between two phenotypes *i* and *j*, we first computed, for each of the 1000 randomly sampled organisms with a given phenotype *i*, all of its L × (A-1) = 2500 single point mutation neighbors. We then determined for all of the resulting 1000 × 2500 neighbors the fraction of neighbors that were viable and had phenotype *j*. We considered this fraction as an estimate of the likelihood that a single point mutation can produce a genotype with phenotype *j* from a genotype with phenotype *i* (i.e., the transition probability *p*_*i*→*j*_). We denote the fraction of non-viable neighbors of the 1000 genotypes with phenotype *i* as *p*_*i*→0_. We note that transition probabilities smaller than 2.5 × 10^−6^ would be equal to zero. We repeated this procedure for all pairs of phenotypes *i* and *j*, and note that transition probabilities need not be symmetric, that is, it may be easier or harder to reach phenotype *j* from phenotype *i* than vice versa.

The asymmetries in transition probabilities are just a consequence of the fact that different phenotypes have different numbers of genotypes that code for them. That is, if a forward mutation produces phenotype *i* from phenotype *j*, then the back mutation produces phenotype *j* from phenotype *i*. Denote as *N*_*i*_ and *N*_*j*_ the number of genotypes with phenotype *i* and *j*, respectively, as *n*_*ij*_ and *n*_*ji*_ the number of mutations from phenotype *i* to phenotype *j* and from *j* to *i*, respectively, and as *A* the size of the alphabet. Then for sequences of length *L* = 100, $p_{i\to j}=\frac{n_{ij}}{(100\left(A-1\right)N_{i}}$ and $p_{j\to i}=\frac{n_{ji}}{(100\left(A-1\right)N_{j}}$. Since *n*_*ij*_ = *n*_*ji*_, $\frac{p_{i\to j}}{p_{j\to i}}=\frac{N_{j}}{N_{i}}$. This result requires no mathematical approximations and does not depend on any assumptions about the topology of the genotype-phenotype map.

Since novel phenotypes arise in evolving populations, we computed the likelihood of reaching phenotype *j* from phenotype *i* in such populations, to test whether the corresponding entry of the transition probability matrix reflect this likelihood (S7 Fig).

## Supporting information

S1 Fig Genotypes with different phenotypes occupy different fractions of genotype space. (PDF)

S2 Fig Sampling phenotype space. (PDF)

S3 Fig Sequence logo. (PDF)

S4 Fig Genotype space characterization. (PDF)

S5 Fig Minimum genome length depends on phenotypic complexity. (PDF)

S6 Fig Phenotypic plasticity varies among phenotypes. (PDF)

S7 Fig Phenotypic transitions probabilities calculated from random sampling and from evolving populations are highly correlated. (PDF)

S1 File Data set. The phenotypes of 1000 × 512 randomly sampled genotypes comprising the whole phenotype space (512 distinct phenotypes for the 9 Boolean operations considered in this study).
(BZ2)

S2 File Configuration files. Avida configuration files and bash scripts used to generate the data set.
(ZIP)

S1 Appendix Self-replication and genotype-phenotype mapping of a digital organism. (PDF)
