# Supplementary material for: The genotype-phenotype map of an evolving digital organism
Source: PLoS Comput Biol. 2017 Feb 27;13(2):e1005414. doi: 10.1371/journal.pcbi.1005414 (PMC5348039; doi:10.1371/journal.pcbi.1005414)
Supplement: S1 Fig — (PDF) [file pcbi.1005414.s001.pdf]

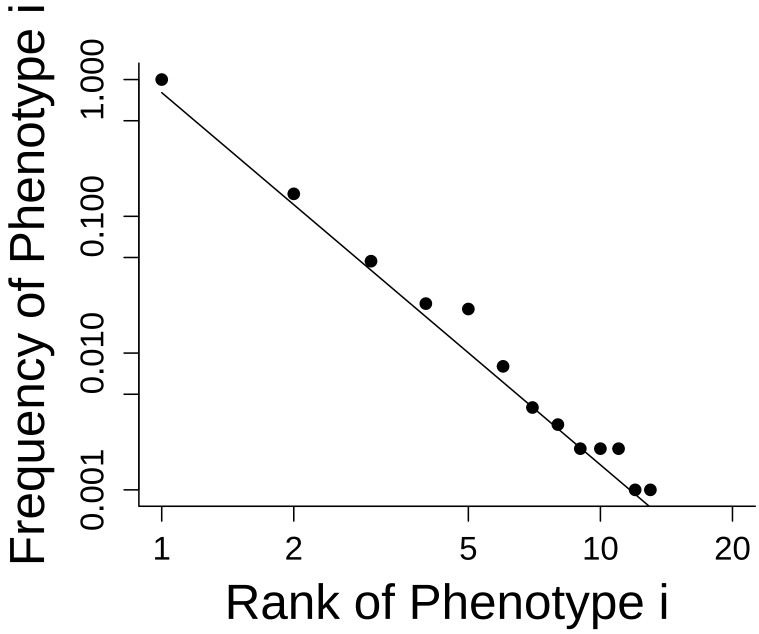

Figure S1: **Genotypes with different phenotypes occupy different fractions of genotype space.** The double-logarithm plot shows the rank of phenotype i (x-axis) versus a measure of its frequency $f(i)$ (y-axis), in genotype space. Data are shown for the 13 most frequent phenotypes, and were obtained by computing the phenotypes of the 2500 $\times$ 1000 1-mutant neighbors of 1000 genotypes uniformly sampled from genotype space. The frequency shown is the number of 1-mutant neighborhoods in which phenotype i appeared at least once. The rank distribution follows a power law.
