# Supplementary material for: The genotype-phenotype map of an evolving digital organism
Source: PLoS Comput Biol. 2017 Feb 27;13(2):e1005414. doi: 10.1371/journal.pcbi.1005414 (PMC5348039; doi:10.1371/journal.pcbi.1005414)
Supplement: S2 Fig — (PDF) [file pcbi.1005414.s002.pdf]

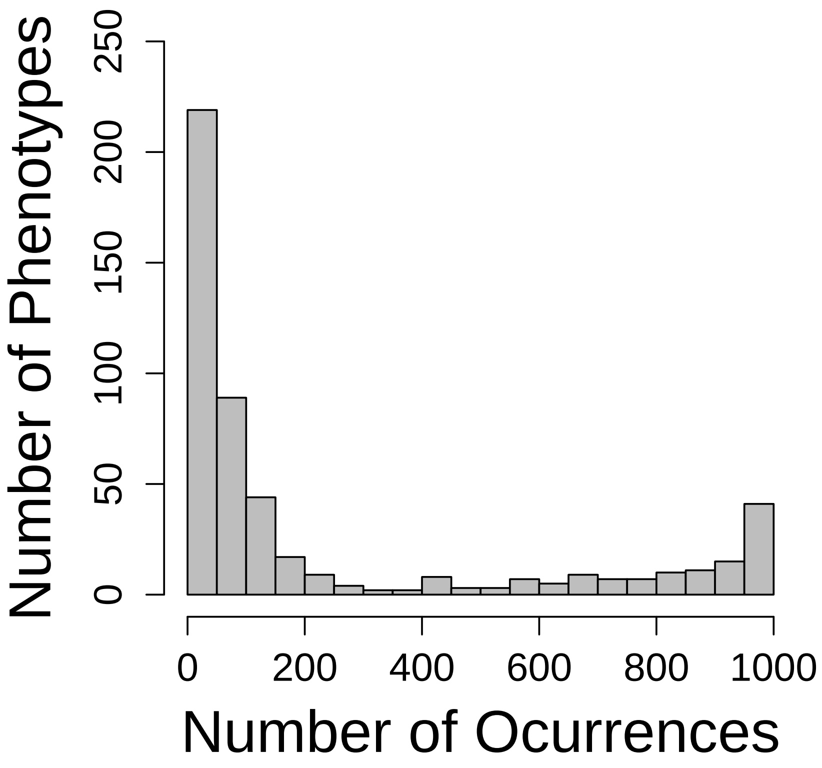

Figure S2: **Sampling phenotype space.** Frequency distribution of all 512 phenotypes discovered in 1000 evolving populations subject to repeated cycles of mutation and selection for performing tasks. Each population was initialized from one of 1000 randomly sampled viable genotypes. Some phenotypes—likely the rarest ones—were only discovered by few populations. Overall, 60% phenotypes were found only by 10% populations (corresponding to the left-most two bars), and only 12% of phenotypes were found by more than 90% of populations (right-most two bars).
