# Supplementary material for: The genotype-phenotype map of an evolving digital organism
Source: PLoS Comput Biol. 2017 Feb 27;13(2):e1005414. doi: 10.1371/journal.pcbi.1005414 (PMC5348039; doi:10.1371/journal.pcbi.1005414)
Supplement: S3 Fig — (PDF) [file pcbi.1005414.s003.pdf]

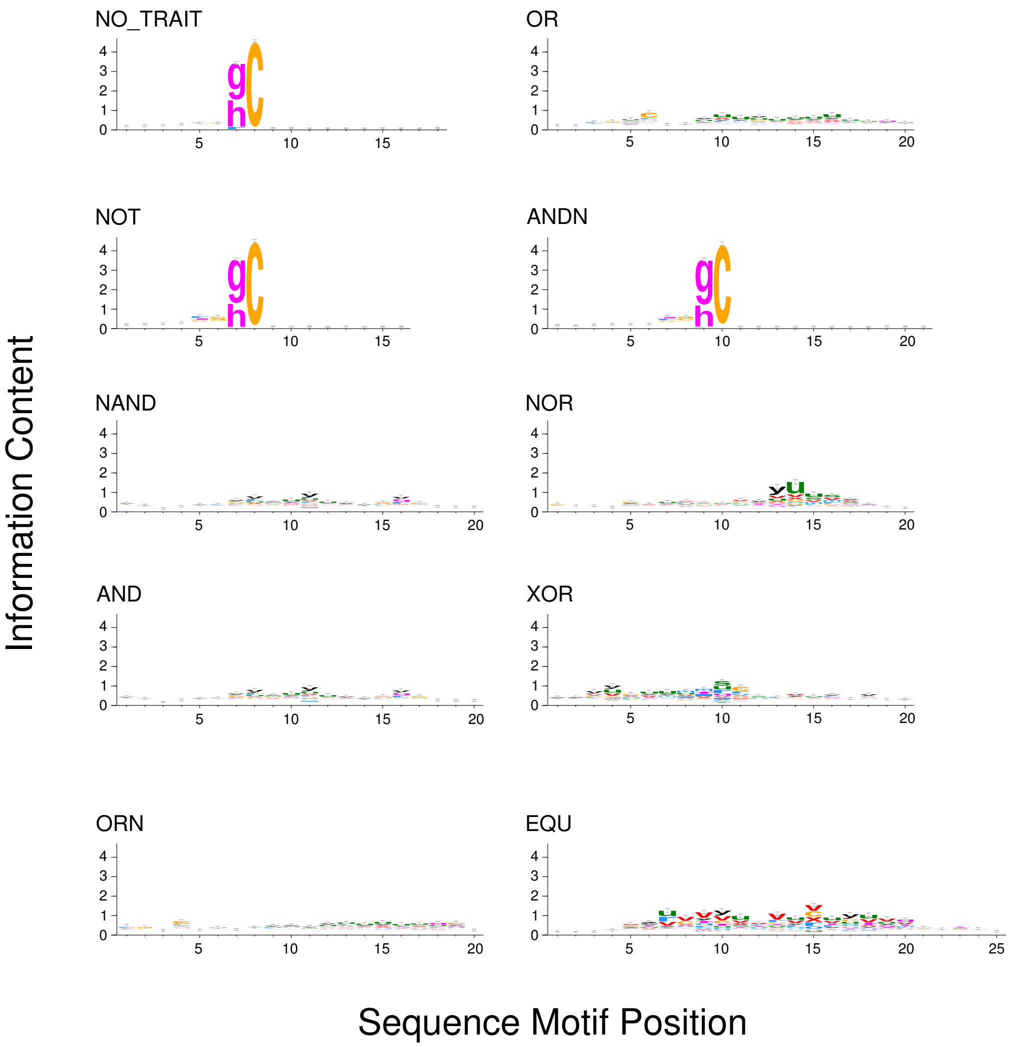

Figure S3: **Sequence logo.** Graphical representation of the genotypic letter sequence motifs detected by the GLAM2 multiple local alignment algorithm (only for merely viable organisms and organisms with single-trait phenotypes). We found a small motif in genotypes encoding the simplest phenotypes. It contains the flow-control operations involved in determining which intructions are going to be read and written. Characters representing the sequence motif are stacked on top of each other for each position in the alignment. The overall height of each stack indicates the sequence conservation at that position (measured in bits), whereas the height of symbols within the stack reflects the relative frequency of the corresponding instruction at that position. No such regions of similarity were found that could reveal the mapping of genotypes into trait-based phenotypes (i.e., functional motifs). We have customized the package RWebLogo to allow for the specific alphabet used in Avida and a color scheme that depicts the five types of instructions germane to this alphabet: no-operators (orange), flow control instructions (magenta), single math instructions (blue), double math instructions (green), copy process instructions (red), and input-output instructions (black).
