# Supplementary material for: The genotype-phenotype map of an evolving digital organism
Source: PLoS Comput Biol. 2017 Feb 27;13(2):e1005414. doi: 10.1371/journal.pcbi.1005414 (PMC5348039; doi:10.1371/journal.pcbi.1005414)
Supplement: S4 Fig — (PDF) [file pcbi.1005414.s004.pdf]

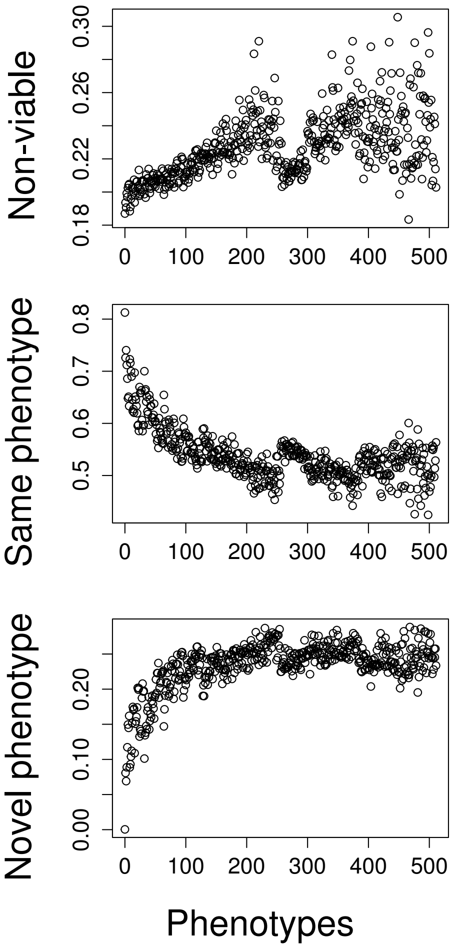

Figure S4: **Genotype space characterization.** Fraction of genotypes that lie in the 1-mutant neighborhood of every organism having a particular phenotype (x-axis): fraction of those genotypes that are non-viable, viable having the same phenotype as the focal phenotype, and viable but having a distinct phenotype. Phenotypes are represented in decimal notation and arranged from left to right in order of increasing complexity (approximately).
