# Supplementary material for: The genotype-phenotype map of an evolving digital organism
Source: PLoS Comput Biol. 2017 Feb 27;13(2):e1005414. doi: 10.1371/journal.pcbi.1005414 (PMC5348039; doi:10.1371/journal.pcbi.1005414)
Supplement: S5 Fig — (PDF) [file pcbi.1005414.s005.pdf]

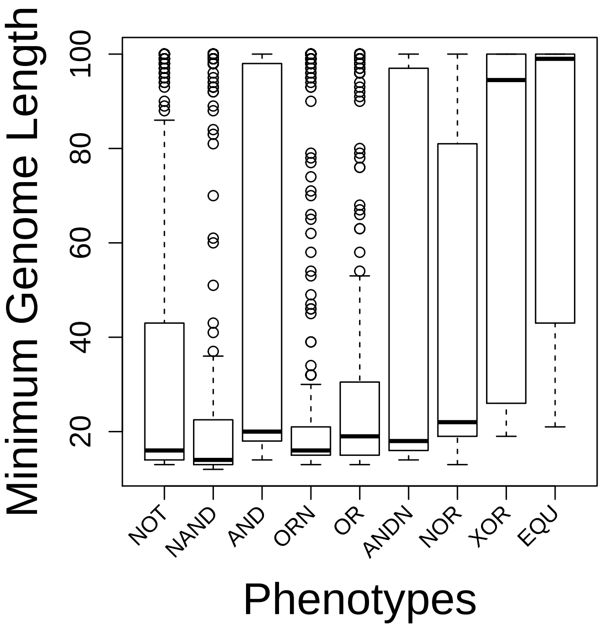

Figure S5: **Minimum genome length depends on phenotypic complexity.** To identify the minimal genome length, we reduced the genome size of all organisms with single-trait phenotypes a step-by-step fashion, removing one randomly chosen instruction at a time while preserving viability and phenotype. Single-trait phenotypes are arranged from left to right in order of increasing complexity (measured as the minimum number of times that a *nand* instruction must be executed to compute the logic function corresponding to that phenotype.
