# Supplementary material for: The genotype-phenotype map of an evolving digital organism
Source: PLoS Comput Biol. 2017 Feb 27;13(2):e1005414. doi: 10.1371/journal.pcbi.1005414 (PMC5348039; doi:10.1371/journal.pcbi.1005414)
Supplement: S6 Fig — (PDF) [file pcbi.1005414.s006.pdf]

Phenoytpic plasticity

Although we computed the phenotype of our 1000×512 randomly sampled genotypes in a constant environment (i.e., we provided the same random sequence of binary input numbers to compute the phenotype of each genotype tested), we also wanted to know to what extent a different environment may elicit additional phenotypes that our constant environment could not. To allow for such "phenotypic plasticity", we computed for the single-trait phenotypes (i.e., the phenotypes of those organisms capable of performing only one logic operation), and for the phenotype whose organisms are merely viable, the phenotype of each genotype in a variable environment. Specifically we provided 1000 distinct random sequences of binary input numbers to each genotype tested. We then quantified the phenotypic plasticity of each genotype as both the number of distinct phenotypes produced by the same genotype (Fig. A) and the Shannon diversity index, which indicates the uncertainty in determining the phenotype of a genotype under different environments (Fig. B). We found that the number of genotypes having more than one phenotype (i.e., the number of plastic genotypes) and the mean value of the Shannon diversity index increase with phenotypic complexity, i.e., with the complexity of the logic functions the organisms are able to compute ($\rho = 0.945$, $n = 10$, $p < 0.001$; $\rho = 0.939$, $n = 10$, $p < 0.001$, respectively). We define this phenotypic complexity as the minimum number of times that a *nand* instruction—the only in struction from the instruction set that is itself a logic operator—must be executed for computing the corresponding logic function.

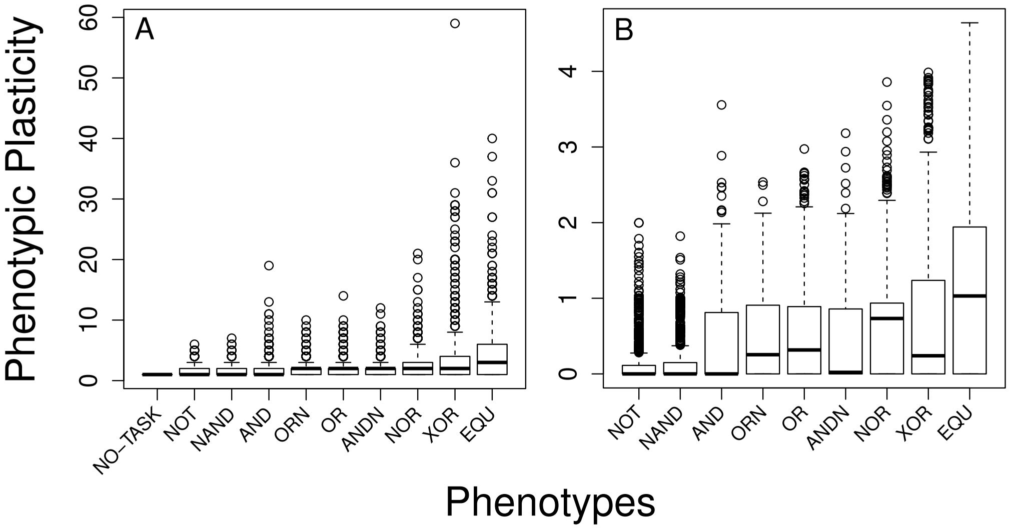

Figure S6: **Phenotypic plasticity varies among phenotypes.** For this analysis, we computed phenotypic plasticity for the 1000 independently sampled genotypes per phenotype. Phenotypes had previously been computed in a constant environment. For each genotype in each set of 1000 genotypes, we computed the phenotype 1000 times in different environments. (Each time a different random sequence of binary numbers was provided as input to the input/output instructions). Then, we computed phenotypic plasticity for each set of 1000 genotypes having the same phenotype in a constant environment as i) the number of distinct phenotypes encoded by the same genotype (A); and ii) the Shannon diversity index $H = \sum_{i=1}^{N} p_i ln(p_i)$, where $N = 512$ indicates the number of distinct phenotypes in phenotype space and p_i indicates the frequency at which each phenotype i ocurred in the set of 1000×1000 genotypes (B). The latter measure indicates how diverse the phenotypes of the set of considered genotypes can be in different environments. It ranges here from 0 (all 1000×1000 genotypes have the same phe notype) to 6.238 (when $p_i = 1/512$ for all $i \in \{1..512\}$). The greater the number of different phenotypes a genotype can encode, and the more equal their abundances are, the greater is the phenotypic plasticity. Plasticity values (vertical axis) are shown for single-trait phenotypes arranged from left to right in order of increasing phenotypic complexity.
