# Supplementary material for: The genotype-phenotype map of an evolving digital organism
Source: PLoS Comput Biol. 2017 Feb 27;13(2):e1005414. doi: 10.1371/journal.pcbi.1005414 (PMC5348039; doi:10.1371/journal.pcbi.1005414)
Supplement: S7 Fig — (PDF) [file pcbi.1005414.s007.pdf]

Phenotypic transitions calculated from evolving populations.

Since novel phenotypes arise in evolving populations, we computed the likelihood of reaching phenotype j from phenotype i in such populations, to test whether the corresponding entry of the transition probability matrix reflect this likelihood. To this end, we computed, for a subset of phenotypes i, the fraction of organisms with a novel phenotype j encountered in 1000 evolving populations, where we initialized each population with our previously sampled 1000 distinct genotypes with phenotype i. In order to record the incidence of novel phenotypes j, we did not allow organisms with phenotype j that originated in a population with phenotype i to replicate. Hence, each population was composed entirely of the offspring of organisms with phenotype i. For each evolving population, we sampled 2500 organisms with distinct genotypes after 1000 updates, and evaluated the phenotypes of the resulting 2.5×10^6 organisms. We note that this is the same number of sampled genotypes that we used to compute the transition probability matrix. Fig. S7 shows the statistical association between the fraction of organisms with phenotype j encountered in evolving populations with phenotype i, and the transition probability from phenotype i to phenotype j.

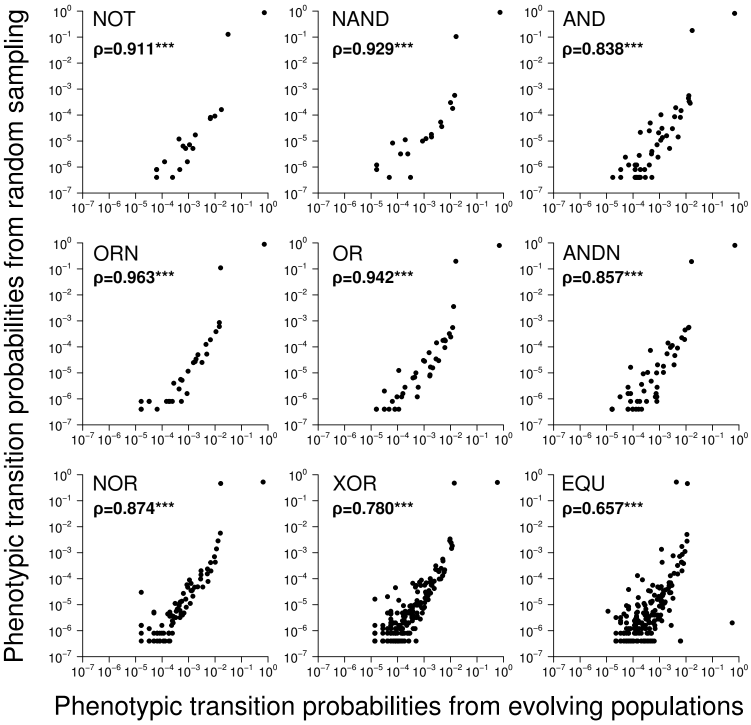

Figure S7: **Phenotypic transition probabilities calculated from random sampling and from evolving populations are highly correlated.** Spearman's rank correlation coefficients between the fraction of organisms with phenotype j encountered from evolving populations with phenotype i and the transition probability from phenotype i to phenotype j. Only organisms with single-trait phenotypes i (indicated as text in the inset) were used in the analysis. Phenotypes are arranged from left to right and top to bottom in order of increasing complexity.
