# Supplementary material for: The genotype-phenotype map of an evolving digital organism
Source: PLoS Comput Biol. 2017 Feb 27;13(2):e1005414. doi: 10.1371/journal.pcbi.1005414 (PMC5348039; doi:10.1371/journal.pcbi.1005414)

Appendix

Self-replication and genotype-phenotype mapping of a digital organism. The entire replication cycle of a digital organism with the smallest genome required to perform the logic operation NAND is depicted in 75 steps. In each step, the genotype of the parent organism is represented on the left as a circular set of 12 instructions (depicted as letters). The growing offspring is represented to the right of its parent. An instruction pointer (black) indicates the instruction that is going to be executed. Three additional pointers—read-head (blue), write-head (red), and flow-head (green)—are used to specify positions in the CPU's memory of the organism. Initially (step 1), the instruction pointer and the three heads are located on the first instruction of this organism's genome (*w: h-alloc*). Arrows indicate the movement of the instruction pointer and write-head from the previous to the current position. Beyond the instructions necessary for copying the genome, the genetic language in Avida contains instructions for storing and manipulating 32-bit numbers in buffers (input-1 and input-2) and registers (AX, BX, and CX). In this cartoon, each binary number is represented as a sequence of 32 boxes, one for each bit. The value of each bit is depicted as a black box if it is 1 and as an white box if it is 0. When an input-ouput instruction is executed (y), the organism outputs the number stored in the BX register. The latest output number provided by the organism after executing an input-output instruction is shown as a sequence of 32 boxes as well. No-operation instructions (i.e., a, b, and c) do not do anything when executed but modify the behavior of the instruction preceding them (e.g., steps 3 and 4). A copy command (instruction v) reads the instruction indicated by the position of the read-head and writes that instruction in an empty

memory space located at the position indicated by the write-head (e.g., step 7). Other instructions (e.g., the move-head instruction represented by the letter g) move the instruction pointer to a position indicated by the flow-head. But, if the no-operation instruction nop-C (c) precedes an instruction that will move a head (e.g., in step 3 c precedes g), the write-head (instead of the instruction pointer) is moved towards the flow-head. For each step, we provide a short description explaining what the instruction to be exectuted will do. The current values of the binary numbers stored in the buffers and registers are shown before the execution of the corresponding instruction. In step 20, the output that will be provided by the execution of an input-output instruction is the result of applying the NAND logic operation on the two input numbers previously stored in the buffers. The result of the NAND logic operation is 0 if and only if the value of the bit of the two inputs is 1, and 1 otherwise. In the next step (21), boxes depicting the output number are colored in red instead of black when the bit value is 1 to highlight the accomplishment of the logic operation). After completing genome replication, the parent organism "buds off" its offspring. Then, the cycle starts again for both organisms independently (each one located in its own memory space).

1.

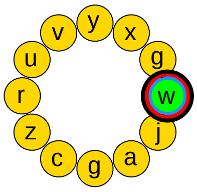

(w) h-alloc

This instruction allocates additional memory for the organism's offspring
(i.e., the equivalent to its genome size),
that will be located from positions 12 to 23.

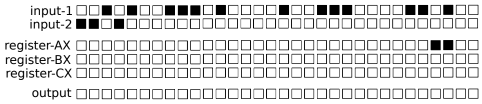

2.

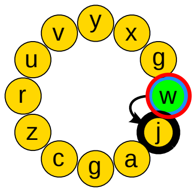

(j) set-flow

This instruction moves the flow-head (green) to the memory position denoted
in the CX register. But, since a nop-A instruction precedes it, the flow-head
will be moved to the position denoted in the AX register (the complement to CX)
(the binary number 1100 stored in the AX register equals 12 in decimal),
which is the first position of the memory allocated to the new offspring.

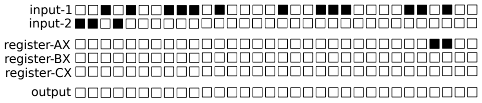

3.

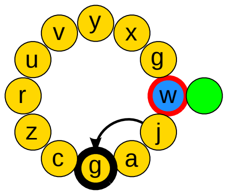

(g) move-head

This instruction will cause the instruction pointer (black) to jump to the position
in memory of the flow-head (green). But, since a nop-C instruction precedes an
instruction that will move a head, the one to be moved will be the head associated to
the non-C instruction (i.e., write-head; in red) instead of the instruction pointer.

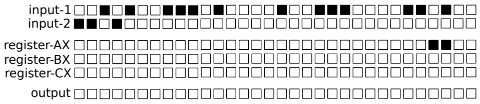

4.

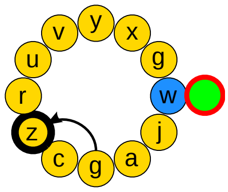

(z) h-search

Since this instruction is not followed by any no-operation instruction,
both BX and CX registers will be set to zero, and the flow-head (green) will
be placed on the instruction immediately following the h-search instruction
(to mark the beginning of the copy loop).

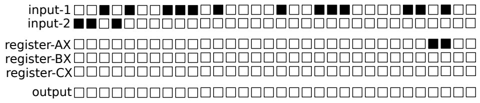

5.

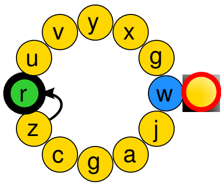

(r) swap

This instruction swaps the contents of the BX register
with its complement (CX register).

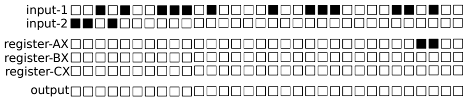

6.

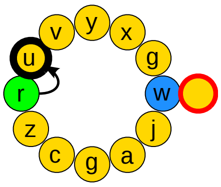

(u) nand

This instruction reads in the contents of the BX and CX registers
and performs a bitwise NAND operation on them.
The result of this operation is placed in the BX register.

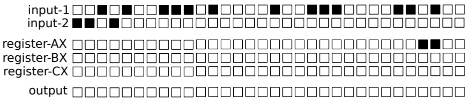

7.

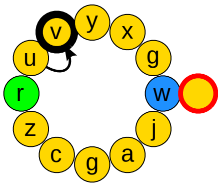

(v) h-copy

This instruction reads in the contents of the organism's memory
at the position of the read-head (blue), copies that
to the position of the write-head (red), and advances both heads.

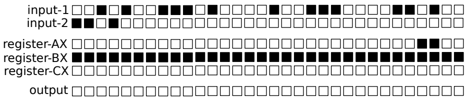

8.

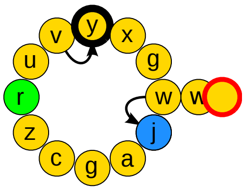

(y) input-output

This instruction takes the contents of the BX register and outputs it,
checking it for any tasks that may have been performed.
It will then place a new input into BX.

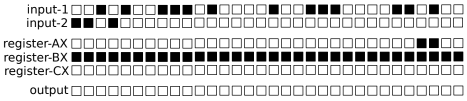

9.

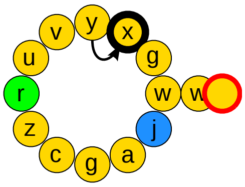

(X) h-divide

This instruction is used by an organism to divide off a finished
offspring located between the read-head and the write-head.
Since the offspring is not finished yet, nothing happens.

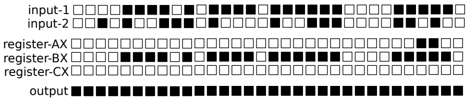

10.

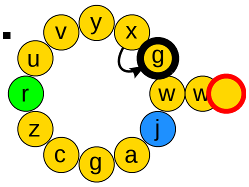

(g) move-head

This instruction will cause the instruction pointer (black)
to jump to the position in memory of the flow-head.

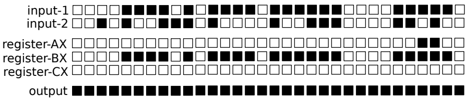

11.

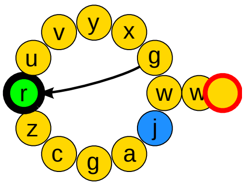

(r) swap

This instruction swaps the contents of the BX register
with its complement (CX register).

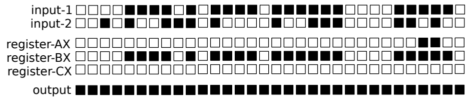

12.

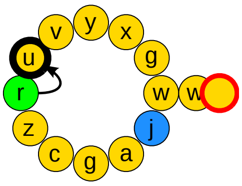

(u) nand

This instruction reads in the contents of the BX and CX registers
and performs a bitwise NAND operation on them.
The result of this operation is placed in the BX register.

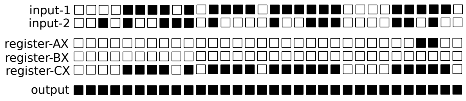

13.

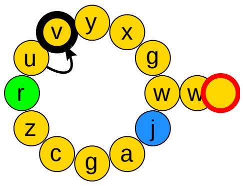

(v) h-copy

This instruction reads in the contents of the organism's memory
at the position of the read-head (blue), copies that
to the position of the write-head (red), and advances both heads).

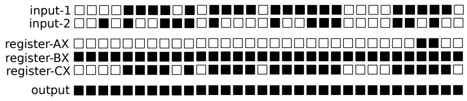

14.

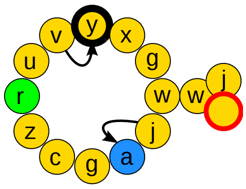

(y) input-output

This instruction takes the contents of the BX register and outputs it,
checking it for any tasks that may have been performed.
It will then place a new input into BX.

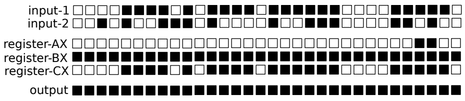

15.

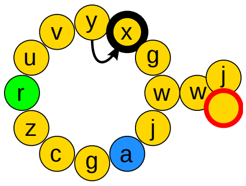

(X) h-divide

This instruction is used by an organism to divide off a finished
offspring located between the read-head and the write-head.
Since the offspring is not finished yet, nothing happens.

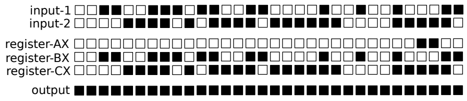

16.

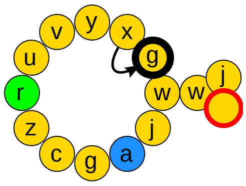

(g) move-head

This instruction will cause the instruction pointer (black)
to jump to the position in memory of the flow-head.

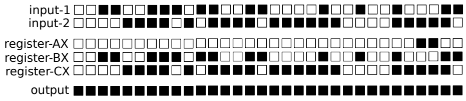

17.

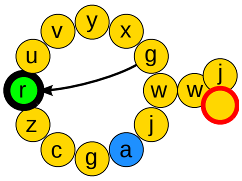

(r) swap

This instruction swaps the contents of the BX register with its complement (CX register).

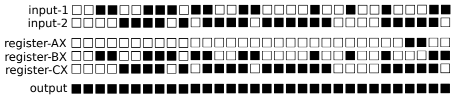

18.

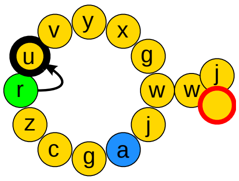

(u) nand

This instruction reads in the contents of the BX and CX registers and performs a bitwise NAND operation on them. The result of this operation is placed in the BX register.

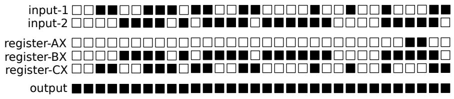

19.

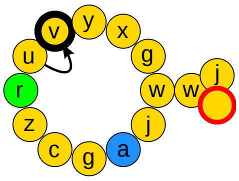

(v) h-copy

This instruction reads in the contents of the organism's memory at the position of the read-head (blue), copies that to the position of the write-head (red), and advances both heads.

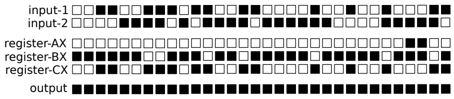

20.

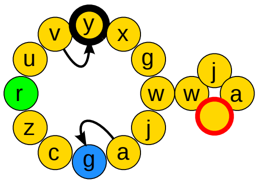

(y) input-output

This instruction takes the contents of the BX register and outputs it, checking it for any tasks that may have been performed. It will then place a new input into BX.

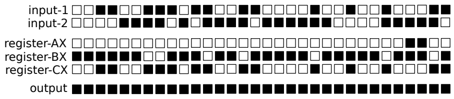

21.

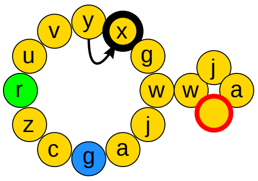

(X) h-divide

This instruction is used by an organism to divide off a finished offspring located between the read-head and the write-head. Since the offspring is not finished yet, nothing happens.

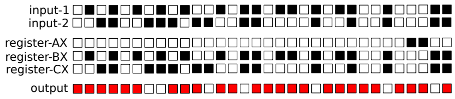

22.

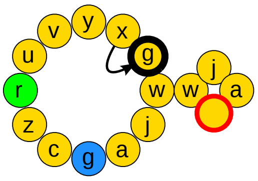

(g) move-head

This instruction will cause the instruction pointer (black) to jump to the position in memory of the flow-head.

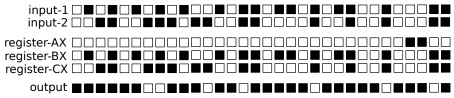

23.

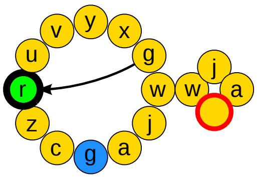

(r) swap
This instruction swaps the contents of the BX register
with its complement (CX register).

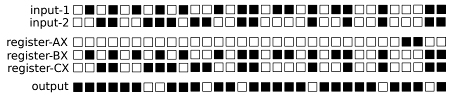

24.

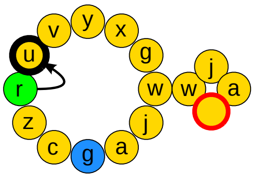

(u) nand
This instruction reads in the contents of the BX and CX registers
and performs a bitwise NAND operation on them.
The result of this operation is placed in the BX register.

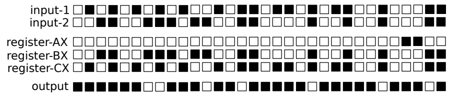

25.

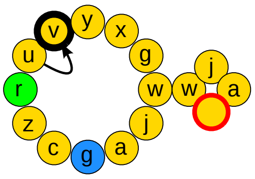

(v) h-copy
This instruction reads in the contents of the organism's memory
at the position of the red-head (blue), copies that
to the position of the write-head (red), and advances both heads.

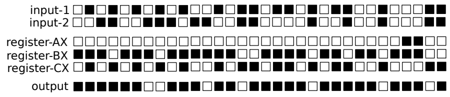

26.

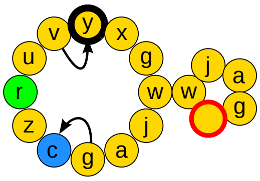

(y) input-output
This instruction takes the contents of the BX register and outputs it,
checking it for any tasks that may have been performed.
It will then place a new input into BX.

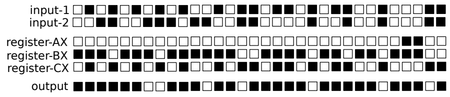

27.

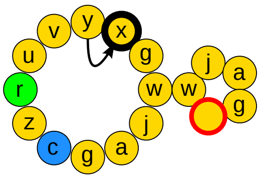

(X) h-divide
This instruction is used by an organism to divide off a finished
offspring located between the read-head and the write-head.
Since the offspring is not finished yet, nothing happens.

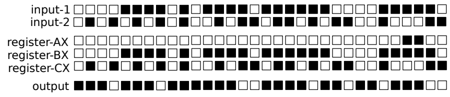

28.

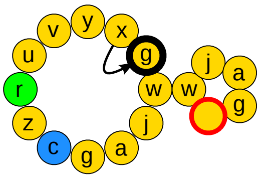

(g) move-head
This instruction will cause the instruction pointer (black)
to jump to the position in memory of the flow-head.

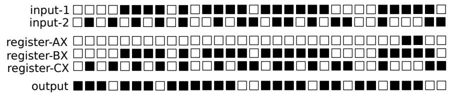

29.

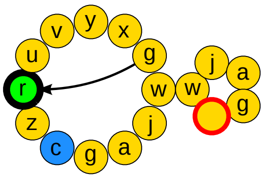

(r) swap

This instruction swaps the contents of the BX register with its complement (CX register).

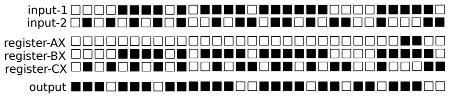

30.

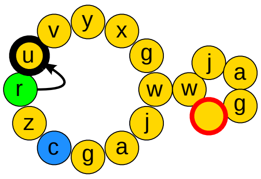

(u) nand

This instruction reads in the contents of the BX and CX registers and performs a bitwise NAND operation on them. The result of this operation is placed in the BX register.

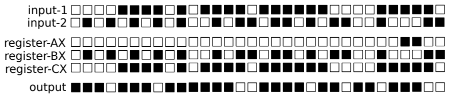

31.

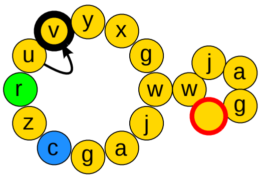

(v) h-copy

This instruction reads in the contents of the organism's memory at the position of the red-head (blue), copies that to the position of the write-head (red), and advances both heads.

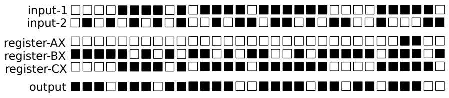

32.

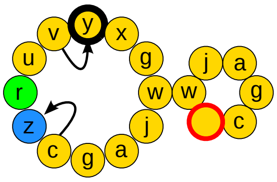

(y) input-output

This instruction takes the contents of the BX register and outputs it, checking it for any tasks that may have been performed. It will then place a new input into BX.

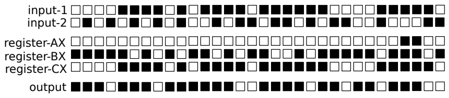

33.

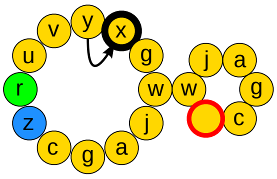

(X) h-divide

This instruction is used by an organism to divide off a finished offspring located between the read-head and the write-head. Since the offspring is not finished yet, nothing happens.

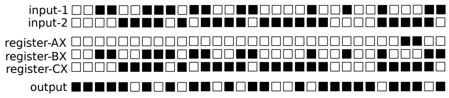

34.

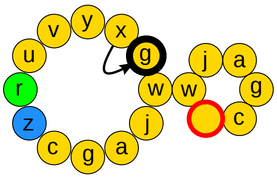

(g) move-head

This instruction will cause the instruction pointer (black) to jump to the position in memory of the flow-head.

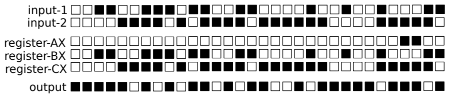

35.

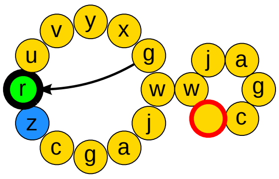

(r) swap
This instruction swaps the contents of the BX register
with its complement (CX register).

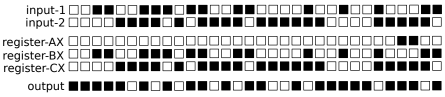

36.

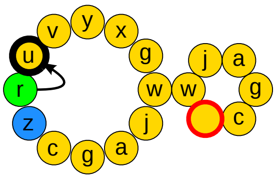

(u) nand
This instruction reads in the contents of the BX and CX registers
and performs a bitwise NAND operation on them.
The result of this operation is placed in the BX register.

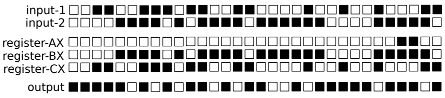

37.

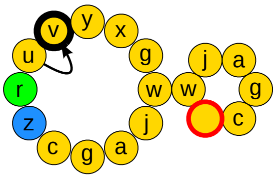

(v) h-copy
This instruction reads in the contents of the organism's memory
at the position of the red-head (blue), copies that
to the position of the write-head (red), and advances both heads.

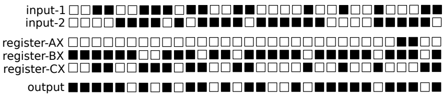

38.

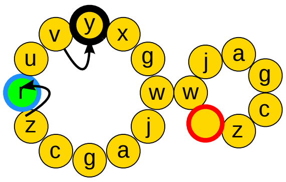

(y) input-output
This instruction takes the contents of the BX register and outputs it,
checking it for any tasks that may have been performed.
It will then place a new input into BX.

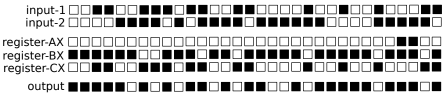

39.

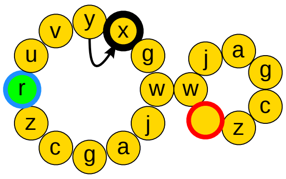

(X) h-divide
This instruction is used by an organism to divide off a finished
offspring located between the read-head and the write-head.
Since the offspring is not finished yet, nothing happens.

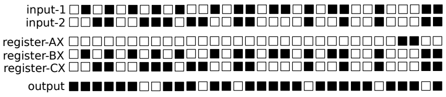

40.

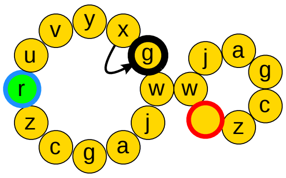

(g) move-head
This instruction will cause the instruction pointer (black)
to jump to the position in memory of the flow-head.

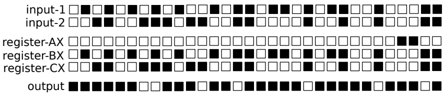

41.

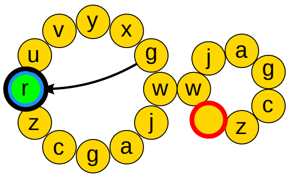

(r) swap
This instruction swaps the contents of the BX register with its complement (CX register).

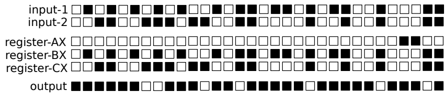

42.

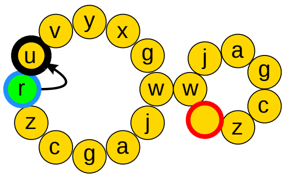

(u) nand
This instruction reads in the contents of the BX and CX registers and performs a bitwise NAND operation on them. The result of this operation is placed in the BX register.

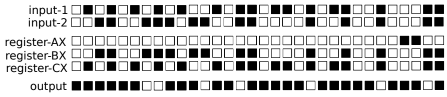

43.

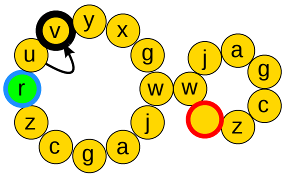

(v) h-copy
This instruction reads in the contents of the organism's memory at the position of the red-head (blue), copies that to the position of the write-head (red), and advances both heads.

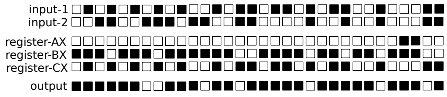

44.

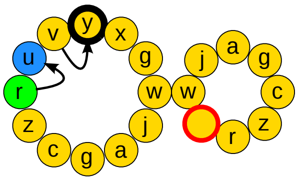

(y) input-output
This instruction takes the contents of the BX register and outputs it, checking it for any tasks that may have been performed. It will then place a new input into BX.

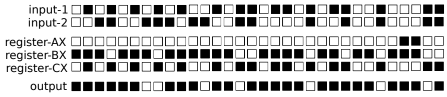

45.

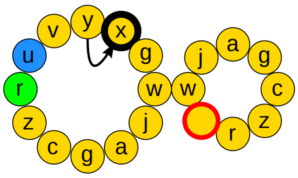

(X) h-divide
This instruction is used by an organism to divide off a finished offspring located between the read-head and the write-head. Since the offspring is not finished yet, nothing happens.

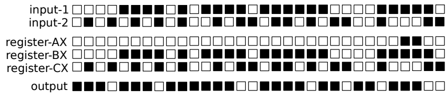

46.

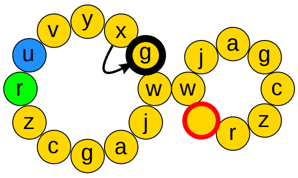

(g) move-head
This instruction will cause the instruction pointer (black) to jump to the position in memory of the flow-head.

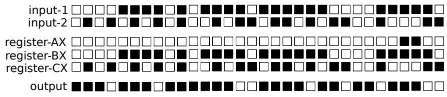

47.

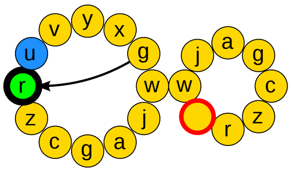

(r) swap
This instruction swaps the contents of the BX register with its complement (CX register).

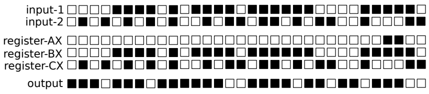

48.

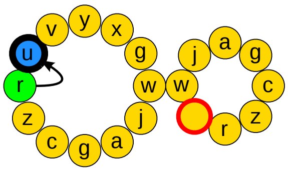

(u) nand
This instruction reads in the contents of the BX and CX registers and performs a bitwise NAND operation on them. The result of this operation is placed in the BX register.

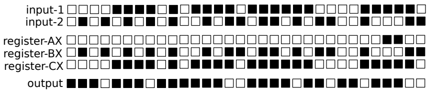

49.

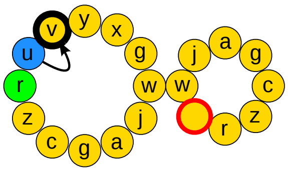

(v) h-copy
This instruction reads in the contents of the organism's memory at the position of the red-head (blue), copies that to the position of the write-head (red), and advances both heads.

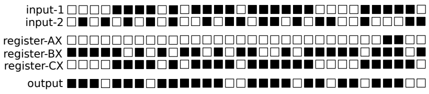

50.

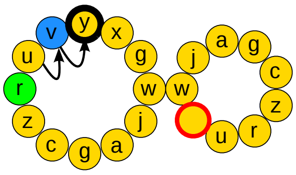

(y) input-output
This instruction takes the contents of the BX register and outputs it, checking it for any tasks that may have been performed. It will then place a new input into BX.

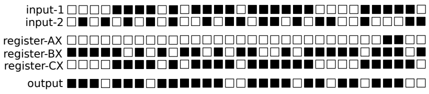

51

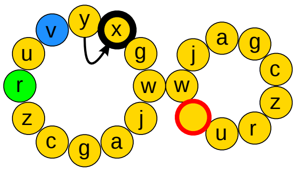

(X) h-divide
This instruction is used by an organism to divide off a finished offspring located between the read-head and the write-head. Since the offspring is not finished yet, nothing happens.

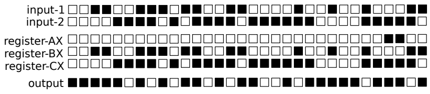

52.

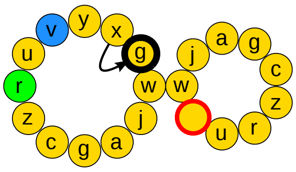

(g) move-head
This instruction will cause the instruction pointer (black) to jump to the position in memory of the flow-head.

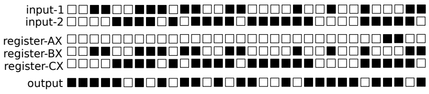

53.

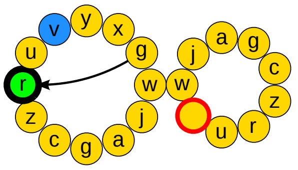

(r) swap

This instruction swaps the contents of the BX register with its complement (CX register).

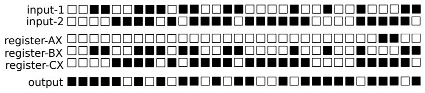

54.

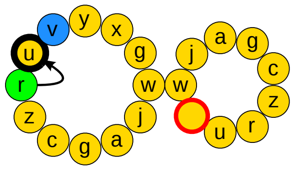

(u) nand

This instruction reads in the contents of the BX and CX registers and performs a bitwise NAND operation on them. The result of this operation is placed in the BX register.

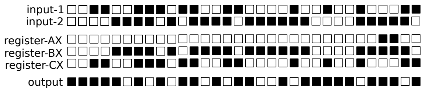

55.

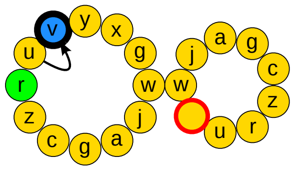

(v) h-copy

This instruction reads in the contents of the organism's memory at the position of the red-head (blue), copies that to the position of the write-head (red), and advances both heads.

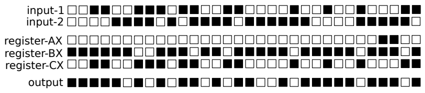

56.

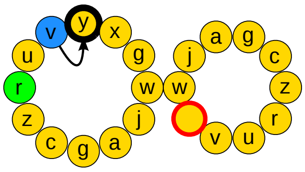

(y) input-output

This instruction takes the contents of the BX register and outputs it, checking it for any tasks that may have been performed. It will then place a new input into BX.

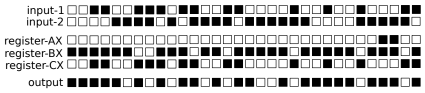

57.

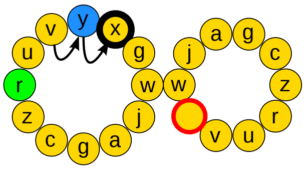

(X) h-divide

This instruction is used by an organism to divide off a finished offspring located between the read-head and the write-head. Since the offspring is not finished yet, nothing happens.

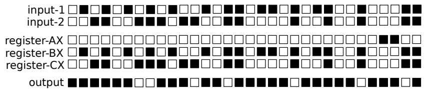

58.

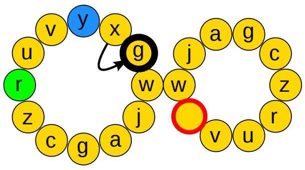

(g) move-head

This instruction will cause the instruction pointer (black) to jump to the position in memory of the flow-head.

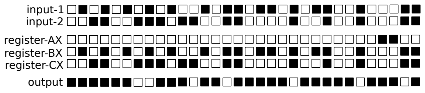

59.

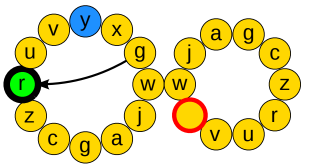

(r) swap
This instruction swaps the contents of the BX register
with its complement (CX register).

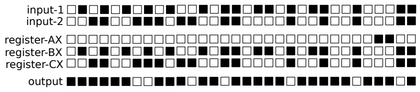

60.

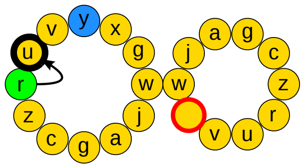

(u) nand
This instruction reads in the contents of the BX and CX registers
and performs a bitwise NAND operation on them.
The result of this operation is placed in the BX register.

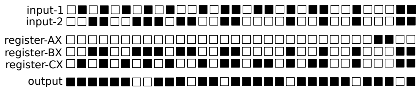

61.

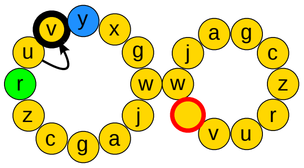

(v) h-copy
This instruction reads in the contents of the organism's memory
at the position of the red-head (blue), copies that
to the position of the write-head (red), and advances both heads.

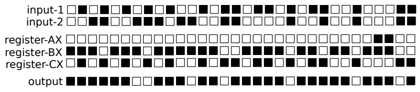

62.

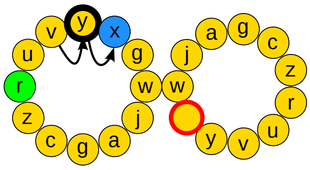

(y) input-output
This instruction takes the contents of the BX register and outputs it,
checking it for any tasks that may have been performed.
It will then place a new input into BX.

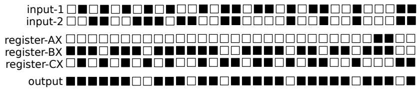

63.

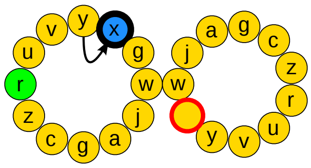

(X) h-divide
This instruction is used by an organism to divide off a finished
offspring located between the read-head and the write-head.
Since the offspring is not finished yet, nothing happens.

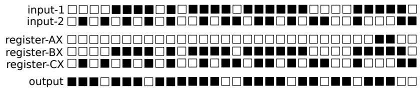

64.

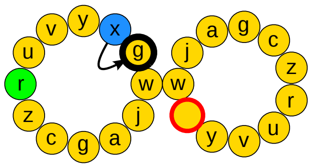

(g) move-head
This instruction will cause the instruction pointer (black)
to jump to the position in memory of the flow-head.

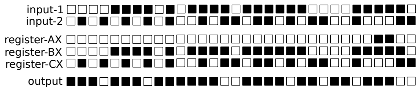

65.

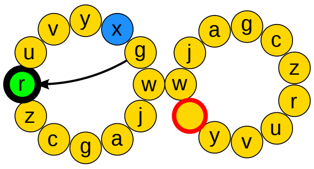

(r) swap

This instruction swaps the contents of the BX register with its complement (CX register).

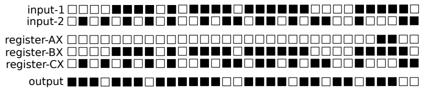

66.

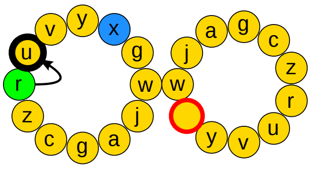

(u) nand

This instruction reads in the contents of the BX and CX registers and performs a bitwise NAND operation on them. The result of this operation is placed in the BX register.

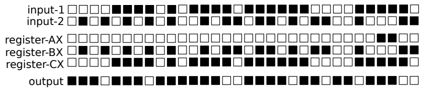

67.

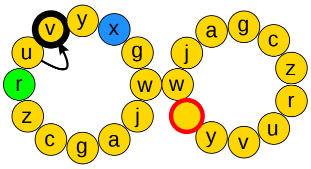

(v) h-copy

This instruction reads in the contents of the organism's memory at the position of the red-head (blue), copies that to the position of the write-head (red), and advances both heads.

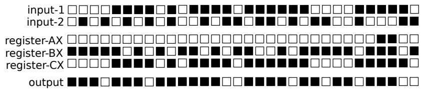

68.

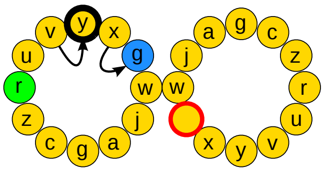

(y) input-output

This instruction takes the contents of the BX register and outputs it, checking it for any tasks that may have been performed. It will then place a new input into BX.

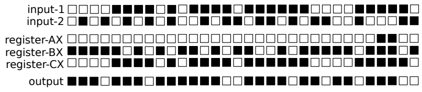

69.

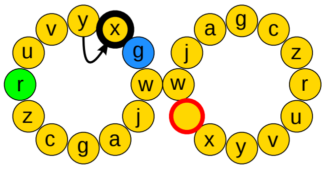

(X) h-divide

This instruction is used by an organism to divide off a finished offspring located between the read-head and the write-head. Since the offspring is not finished yet, nothing happens.

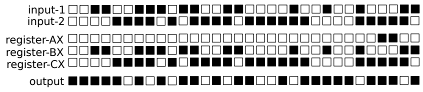

70.

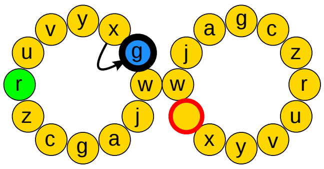

(g) move-head

This instruction will cause the instruction pointer (black) to jump to the position in memory of the flow-head.

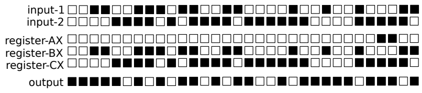

71.

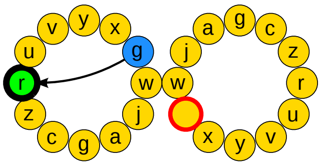

(r) swap
This instruction swaps the contents of the BX register with its complement (CX register).

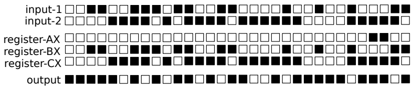

72.

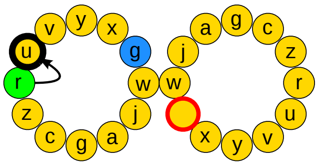

(u) nand
This instruction reads in the contents of the BX and CX registers and performs a bitwise NAND operation on them. The result of this operation is placed in the BX register.

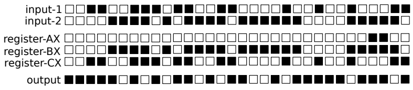

73.

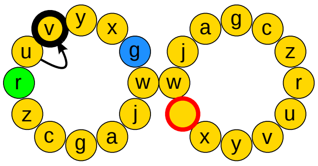

(v) h-copy
This instruction reads in the contents of the organism's memory at the position of the red-head (blue), copies that to the position of the write-head (red), and advances both heads (the read-head to position 12 (first position of the offspring) and the write-head to position 0 (the first one of the parent).

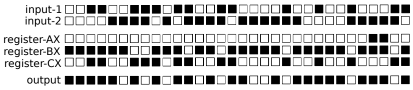

74.

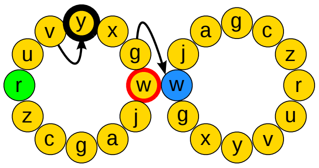

(y) input-output
This instruction takes the contents of the BX register and outputs it, checking it for any tasks that may have been performed. It will then place a new input into BX.

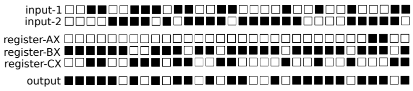

75.

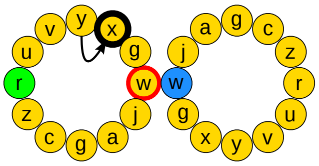

(X) h-divide
This instruction is used by an organism to divide off a finished offspring located between the read-head and the write-head. Since the offspring is actually finished (all its memory positions have been filled), both organisms (each one located in its own memory space) start the process again.

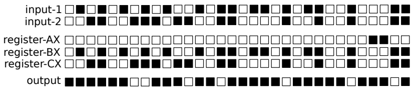

76.

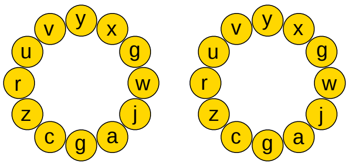

Supplement: S1 Appendix — (PDF) [file pcbi.1005414.s010.pdf]
